# Dust Storms and Emergency Department Visits in 3 Southwestern States Using NWS Storm Reports

**DOI:** 10.1001/jamanetworkopen.2024.57666

**Published:** 2025-02-12

**Authors:** Xiaping Zheng, Howard H. Chang, Stefanie T. Ebelt, Rohan D’Souza, Kirk Hohsfield, James L. Crooks

**Affiliations:** 1 Department of Biostatistics and Bioinformatics, Rollins School of Public Health, Emory University, Atlanta, Georgia; 2 Gangarosa Department of Environmental Health, Rollins School of Public Health, Emory University, Atlanta, Georgia; 3 Department of Epidemiology, Colorado School of Public Health; 4 Division of Biostatistics and Bioinformatics, National Jewish Health, Denver, Colorado

## Abstract

**Importance:**

Dust storms are projected to increase with climate change. The short-term health outcomes associated with dust storms in the US are not well characterized, especially for morbidity outcomes.

**Objective:**

To estimate associations between dust storms and diagnosis-specific emergency department (ED) visits during 2005 to 2018.

**Design, Setting, and Participants:**

In this cross-sectional study using a time-stratified case-crossover design, short-term associations between dust storms and ED visits were estimated at the zip code level using conditional Poisson analysis with adjustment for meteorology and within-month trends. Same-day dust storm events and storm events within a lag period of up to 7 days were considered. State-wide patient-level ED visit records acquired from 3 state health departments (Arizona, California, and Utah) were analyzed. Data were analyzed between April 21 and November 12, 2024.

**Exposures:**

Dust storm events were reported by the US National Weather Service and assigned to each patient zip code that had at least a 5% areal overlap with the National Weather Service forecast zone.

**Main Outcomes and Measures:**

Patient-level ED visits for asthma, chronic obstructive pulmonary disease, culture-negative pneumonia, congestive heart failure (CHF), cerebrovascular disease, ischemic heart disease, and visits due to motor vehicle accidents.

**Results:**

The analysis included 33 500 ED visits among the outcomes of interest (5717 children aged 0-17 years [17.1%] and 11 150 adults aged >65 years [33.3%]; 17 394 male [51.9%] and 16 104 female [48.1%]; 2829 Black [8.4%] and 22 537 White [67.2%]; 9256 Hispanic [27.6%]) and 206 dust-impacted zip codes. The strongest associations between dust storms and ED visits were found for asthma (lag 0-2 relative risk [RR], 1.06; 95% CI, 1.01-1.11; *P* = .03), culture-negative pneumonia (lag 0-7 RR, 1.06; 95% CI, 1.02-1.10; *P* = .002), CHF (lag 0-7 RR, 1.06; 95% CI, 1.01-1.10; *P* = .01), and motor vehicle accidents (lag 0 RR, 1.13; 95% CI, 1.04-1.23; *P* = .003). Associations of dust storm exposure with ischemic heart disease were mostly protective (eg, lag 0-2 RR, 0.89; 95% CI, 0.84-0.95; *P* < .001). Associations of dust storm exposure with risk of ED visits for CHF and motor vehicle accidents were robust against adjustment for ambient ozone (eg, CHF: RR, 1.08; 95% CI, 1.03-1.13; *P* = .003) and nitrogen dioxide (eg, CHF: RR, 1.08; 95% CI, 1.03-1.13; *P* = .003) air pollution.

**Conclusions and Relevance:**

In this study, dust storms were positively associated with ED visits for asthma, pneumonia, heart failure, and motor vehicle accidents. These findings contribute to our understanding of the association of dust storms with morbidity in the US and potential outcomes under a changing climate.

## Key Points

**Question:**

Are dust storms associated with the rate of cardiopulmonary and motor vehicle accident emergency department visits in the US Southwest?

**Findings:**

In this cross-sectional study of 33 500 emergency department visits, dust storms were positively associated with visits for asthma, culture-negative pneumonia, and motor vehicle accidents but negatively associated with ischemic heart disease.

**Meaning:**

These findings suggest that dust storms may be a significant contributor to increased health care utilization under a warming climate.

## Teaser

This cross-sectional study investigates the association of dust storm exposure, as reported by the National Weather Service (NWS), with emergency department visits by diagnosis in Arizona, California, and Utah.

## Introduction

Dust storms, which are meteorological phenomena characterized by strong winds that lift soil and particulates into the air, pose substantial environmental and health risks, particularly in arid and semiarid regions. These events are influenced by weather and climatic variability and by human activities, such as land use changes and agricultural practices.^1,2^ Evidence that dust storms have increased in frequency and intensity in the recent past varies by region.^1,3,4,5^ Evidence that dust storms will increase in the future due to global climate change also varies by region^6,7,8,9^ given that increasing temperatures and prolonged periods of drought enhance the conditions favorable to dust storm formation in some areas.

The US Southwest, comprising Arizona, California, Nevada, New Mexico, and Utah, frequently experiences dust storms. These intense storms increase levels of airborne fine particulate matter pollution (PM_2.5_ and PM_10_, particulate matter with aerometric diameters <2.5 μm and <10 μm, respectively). PM_2.5_ and PM_10_ are known to penetrate the lungs, with the smallest particles able to enter the bloodstream, leading to a range of adverse health problems.^10,11^

Understanding health outcomes associated with these dust storms is crucial for developing effective public health interventions and enhancing emergency preparedness and response strategies. Published work from East Asia,^12,13,14,15,16,17,18,19,20,21,22,23,24,25,26,27,28,29,30,31,32^ southern Europe,^33,34,35,36,37,38,39,40,41,42,43,44^ the Middle East,^45,46,47,48,49^ and Australia^50,51,52,53^ has found associations between dust storms (or airborne dust and sand events more broadly) and increases in risk of mortality,^3,21,22,25,26,33,34,36,37,38,42,44,47,54,55^ hospitalization, intensive care unit (ICU) admissions,^40,48,56,57^ and emergency department (ED) visits^19,52,58^ for respiratory, cardiovascular, and other causes, as well as reduced lung function and infectious diseases. Prior work from around the world has also found associations between dust storms and motor vehicle accidents,^59,60,61,62^ and model-based warning systems for drivers have been developed in some locations.^63,64^

Despite the recognized risk of dust storms, there is a relative paucity of research quantifying direct health outcomes associated with dust storms in the US. Previous work has found dust storms to be positively associated with nonaccidental mortality^3^ and total and respiratory ICU admissions^57^ over subsequent days across the Western US, motor vehicle accidents in 3 California counties,^62^ and, less certainly, Valley fever in Arizona and California.^65,66^ However, to our knowledge, only a single study in the US^67^ has investigated associations with ED visits, and it was limited to zip codes located near PM monitors in the Interagency Monitoring of Protected Visual Environments (IMPROVE) network, which measures air quality in US national parks, and to days when IMPROVE monitors are collecting data (roughly every third day). ED visits constitute an important indicator of acute health problems. The ED is the only component in the US health care system that is accessible at any time of day, and it plays important roles in disaster response.^68,69,70^ ED visits are more common than other administrative outcomes, such as hospitalizations and ICU admissions. In 2017, there were 144.8 million ED visits in the US, costing a total of $76.3 billion.^71^ Because EDs provide treatment regardless of the ability of patients to pay, these settings often serve as the safety net for underresourced populations with care-seeking barriers.^72,73,74^ Furthermore, the ED setting is noted as being a more sensitive and real-time indicator than mortality.^75,76^

This study aimed to bridge the gap in knowledge about dust storms and ED visits in the US. We hypothesized that dust storms would have positive associations with certain respiratory and cardiovascular outcomes, as well as motor vehicle accidents. This study investigated their associations across 3 Southwestern states where dust storms are relatively frequent using dust storm data with greater spatial coverage and temporal completeness than prior ED work, with a resulting increase in scope and power.

## Methods

This cross-sectional study was approved by the Emory University Institutional Review Board, with a waiver of consent. The waiver of consent was granted given that this research was deemed to involve no more than minimal risk, could not practicably be carried out without the requested waiver, and involved use of identifiable private information and could not practicably be carried out without using such information in an identifiable format and given that the waiver would not adversely affect the rights and welfare of participants. The study follows the Strengthening the Reporting of Observational Studies in Epidemiology (STROBE) reporting guideline for cross-sectional studies.

### Study Area and Population

This study focused on 3 states in the US Southwest: Arizona, California, and Utah. These states were selected based on their high incidence of dust storms and availability of comprehensive health data. The study period spans from 2005 to 2018 (Arizona: July 1, 2010, to December 31, 2018; California: January 1, 2005, to December 31, 2018; Utah: January 1, 2005, to December 31, 2016). The difference in time periods by state is due to differences in data availability from the 3 health departments.

### Dust Storm Events

Data on dust storm occurrences were sourced from the US National Weather Service (NWS) Storm Events Database^77^ and are localized to NWS Weather Forecast Zones (WFZs). In the Eastern US, WFZs tend to be coterminous with counties, but in the Western US, they tend to be smaller than counties and more closely follow topographic features. All storm events with EVENT_TYPE listed as *Dust Storm* were included.

The storm dataset includes information on the timing, duration, and geographical extent of each dust storm, as well as the NWS regional office generating the dust storm report. Where multiple dust storm events were reported on the same day in the same WFZ, they were treated as a single dust storm event. When a single dust storm was reported to last into a second day, it was treated as 2 dust storms. Each dust storm event was georeferenced to ED patient residential zip codes based on a minimum 5% areal overlap with NWS forecast zones. A map of these zip codes is found in eFigure 1 in Supplement 1.

### Emergency Department Visits

Patient-specific ED visit records were obtained from the 3 state health departments, constituting complete capture during each state’s study years. However, only zip codes overlapping NWS WFZs in which at least 1 dust storm was reported during the study period were included in the study. Many of these zip codes, especially those in Utah, were in rural areas and thus contributed few ED visits.

ED visit records included the date of visit, primary and secondary *International Classification of Diseases, Ninth Revision *(*ICD-9*) and *International Statistical Classification of Diseases and Related Health Problems, Tenth Revision *(*ICD-10*) diagnoses, patient demographics (age, sex, race, and ethnicity), and zip code of residence. Race and ethnicity data were taken from state health department administrative databases, where this information was taken from hospital billing record data. Categories varied but included American Indian or Native Alaskan, Asian, Black, Native Hawaiian or Other Pacific Islander, White, and multiracial; numbers are reported only for Black and White participants in the demographic table because other categories were small and categories were not consistent across state or time. Race and ethnicity are reported to provide a demographic description of the study population. The health outcomes analyzed were chosen to reflect conditions previously found to be associated with or thought to be exacerbated by dust exposure, including respiratory conditions (asthma [*ICD-9*: 493; *ICD-10*: J45], chronic obstructive pulmonary disease [COPD; *ICD-9*: 491, 492, and 496; *ICD-10*: J41-J44], and culture-negative pneumonia [*ICD-9*: 485-486; *ICD-10*: J18]), cardiovascular conditions (congestive heart failure [*ICD-9*: 428; *ICD-10*: I42, I50-I51], ischemic heart disease [*ICD-9*: 410-414; *ICD-10*: I20-I25], and cerebrovascular disease [*ICD-9*: 430-434, 436-438; *ICD-10*: I60-I69]), and motor vehicle accidents (*ICD-9*: E810-E819; *ICD-10*: V87-V89). Outcomes were defined based on primary *ICD-9* or *ICD-10* diagnosis code, except for motor vehicle accidents, which were defined based on secondary diagnosis codes given that no primary codes were observed for this outcome.

### Meteorology and Air Pollution

ED visits may be influenced by other air pollutant exposures aside from dust storms in ways that may have confounded our main association. Daily ambient air pollution data for nitrogen dioxide (NO_2_; 1-h maximum), ozone (O_3_; 8-h maximum) and PM_2.5_ (24-h mean) concentrations at a 1 km × 1 km spatial resolution were obtained from the Socioeconomic Data and Applications Center (SEDAC),^78^ and the spatial mean was found to the zip code level. Air pollution data were available only for 2005 to 2016, and therefore all analyses that included these air pollutant variables excluded the years 2017 and 2018.

PM_10_ data were not available from SEDAC. However, much of the airborne dust in dust storms is in the form of PM_10_, and it has been shown^3,57^ that ambient PM_10_ concentrations tend to be highly correlated with dust storm events. Therefore, controlling for PM_10_ would be inappropriate.

### Exposure Assessment

Binary indicators for the presence of dust storm events were assigned to zip code- and cause-specific daily ED visit counts. We considered exposures defined by whether a dust storm occurred on the same day (lag 0), over the last 2 days (lag 0-2), over the last 5 days (lag 0-5), or over the last week (lag 0-7). Days falling on the same day of week in the same month but without a storm event were treated as matched control days within a stratum. This design controls for time-invariant confounders and is particularly suited for acute environmental exposures, such as dust storms. Only strata that had at least 1 dust storm were included in the analysis because strata without any event could not contribute to the estimation of dust storm outcomes.

### Statistical Analysis

Time-stratified case-crossover conditional quasi-Poisson regression models were used to estimate the relative risk (RR) of the outcome (daily ED visit counts) associated with exposure to dust storms. ED visit counts on control days served as the control population. We controlled for temperature using a combination of natural splines with 5 degrees of freedom each for mean temperature on the exposure day and 3-day moving mean temperature on the days after the exposure day. We controlled for dew point temperature in the same way. Strata-specific intercepts automatically controlled for temporally invariant factors within each zip code, as well as smoothly but slowly varying factors in time. We accounted for shorter-term time trends by including a temporal spline in the model with 4 degrees of freedom per year. Crude models with only dust storm term and strata-specific intercepts were also estimated. The level of significance was set at α = .05 unless otherwise stated, and *P* values were 2-sided. Analyses were performed using R statistical software version 4.4.1 (R Project for Statistical Computing) and SAS statistical software version 9.4 (SAS Institute). Data were analyzed between April 21 and November 12, 2024.

#### Attributable Fraction

Based on the distribution of coefficients estimated by our main model, we simulated 5000 total ED visit counts under the observed distribution of dust storms and under a scenario with the dust storm variable set to 0, which we label N_dust,_*_i_* and N_nodust,_*_I_,* respectively, for replicate *i*. The attributable fraction (*AF*) for replicate *i* expressed as a percentage was then *AF_i_* = 100% × [1 − (N_nodust,_*_i_*/N_dust,_*_i_*)]. From the resulting distribution, we calculated 2.5th, 50th, and 97.5th percentiles of the attributable fraction. Due to the structure of our models, we calculated this fraction with respect to the number of ED visits encompassed by the set of zip code-days used in our models, not all ED visits.

#### Sensitivity Analyses

Sensitivity analyses were conducted to assess the robustness of findings across different model specifications. These analyses included (1) varying the overlap percentage used to assign dust storms to zip codes from 5% to 10% and 20%, which changes the balance between sample size and exposure accuracy (in particular, by switching some dust storm exposure values from 1 to 0); (2) defining outcomes based on both primary and secondary *ICD-9* and *ICD-10* diagnosis codes as opposed to primary only; (3) varying the degrees of freedom in spline functions used to model temporal trends (degrees of freedom/y = 4, 8, or 12); (4) controlling air pollutants (O_3_, NO_2_, and PM_2.5_) in models (recall that these models do not use ED visits and dust storms from 2017 to 2018 owing to data availability), and (5) comparing results between outpatient ED visits and inpatient ED visits resulting in hospitalization.

## Results

### Descriptive Statistics

The main analyses encompassed 206 reported days of dust storms and 129 506 ED visits by patients residing in zip codes with 5% areal overlap with NWS forecast zones in which dust storms are reported (eFigure 1 in Supplement 1). Of the ED visits, 33 500 visits fell into time strata used in our analyses (5717 children aged 0-17 years [17.1%] and 11 150 adults aged >65 years [33.3%]; 17 394 male [51.9%] and 16 104 female [48.1%]; 2829 Black [8.4%] and 22 537 White [67.2%]; 9256 Hispanic [27.6%]) (Table 1).

**Table 1.** ED Visit Outcomes by Demographic Characteristic

Characteristic	ED visits, No. (%)^a^							
	Asthma (n = 6734)	COPD (n = 3768)	CNP (n = 6303)	CHF (n = 2762)	CVD (n = 2917)	IHD (n = 4263)	MVA (n = 6753)	Total (N = 33 500)^b^
Age, y								
0-17	2920 (43.4)	1 (<0.1)	1661 (26.4)	3 (0.1)	20 (0.7)	1 (<0.1)	1111 (16.5)	5717 (17.1)
18-29	1051 (15.6)	19 (0.5)	382 (6.1)	15 (0.5)	30 (1)	17 (0.4)	2135 (31.6)	3649 (10.9)
30-44	1137 (16.9)	108 (2.9)	714 (11.3)	156 (5.6)	163 (5.6)	205 (4.8)	1605 (23.8)	4088 (12.2)
45-64	1100 (16.3)	1515 (40.2)	1326 (21)	803 (29.1)	856 (29.3)	1756 (41.2)	1364 (20.2)	8720 (26.0)
≥65	494 (7.3)	2125 (56.4)	2098 (33.3)	1780 (64.4)	1846 (63.3)	2284 (53.6)	523 (7.7)	11 150 (33.3)
Unknown	32 (0.5)	0	122 (1.9)	5 (0.2)	2 (0.1)	0	15 (0.2)	176 (0.5)
Sex								
Female	3447 (51.2)	2071 (55.0)	3013 (47.8)	1136 (41.1)	1369 (46.9)	1567 (36.8)	3501 (51.8)	16 104 (48.1)
Male	3287 (48.8)	1697 (45.0)	3290 (52.2)	1626 (58.9)	1547 (53.0)	2695 (63.2)	3252 (48.2)	17 394 (51.9)
Unknown	0	0	0	0	1 (<0.1)	1 (<0.1)	0	2 (<0.1)
Race								
Black	1059 (15.7)	220 (5.8)	369 (5.9)	220 (8.0)	169 (5.8)	197 (4.6)	595 (8.8)	2829 (8.4)
White	3696 (54.9)	3166 (84.0)	4299 (68.2)	2032 (73.6)	2172 (74.5)	3315 (77.8)	3857 (57.1)	22 537 (67.3)
Other^c^	1894 (28.1)	349 (9.3)	1551 (24.6)	479 (17.3)	542 (18.6)	701 (16.4)	2150 (31.8)	7666 (22.9)
Unknown	85 (1.3)	33 (0.9)	84 (1.3)	31 (1.1)	34 (1.2)	50 (1.2)	151 (2.2)	468 (1.4)
Ethnicity								
Hispanic	2491 (37.0)	380 (10.1)	1986 (31.5)	617 (22.3)	595 (20.4)	797 (18.7)	2390 (35.4)	9256 (27.6)
Non-Hispanic	4154 (61.7)	3342 (88.7)	4235 (67.2)	2118 (76.7)	2294 (78.6)	3419 (80.2)	4199 (62.2)	23 761 (70.9)
Unknown	89 (1.3)	46 (1.2)	82 (1.3)	27 (1.0)	28 (1.0)	47 (1.1)	164 (2.4)	483 (1.4)

Abbreviations: CHF, congestive heart failure; COPD, chronic obstructive pulmonary disease; CNP, culture-negative pneumonia; CVD, cerebrovascular disease; ED, emergency department; IHD, ischemic heart disease; and MVA, motor vehicle accident.

^a^ This demographic table provides patient ED visit outcomes during the study period for 3 southwestern US states: Arizona (July 1, 2010, to 2018), California (2005-2018), and Utah (2005-2016). Analysis was restricted to zip codes with at least a 5% area overlap with a National Weather Service forecast zone in which at least 1 dust storm was reported. Outcomes were based on the primary diagnosis, except for motor vehicle accidents, for which both primary and secondary diagnoses were used.

^b^ Of the 33 500 ED visit outcomes, 10 are double-counted because they include a primary respiratory or cardiovascular code and a secondary motor vehicle accident code.

^c^ Other race could include American Indian or Native Alaskan, Asian, Native Hawaiian or Other Pacific Islander, multiracial, or refused to answer. However, these categories differed by state and shifted over time.

Cause-specific counts of overall ED visits in the study period in the states under investigation are presented in eTable 1 in Supplement 1. Table 2 presents cause-specific counts of those ED visits falling into time strata used in our analyses. Among our studied outcomes, the largest numbers of ED visits falling into time strata were for motor vehicle accidents (6753 visits [20.2%]) and asthma (6734 visits [20.1%]), followed closely by culture-negative pneumonia (6303 visits [18.8%]). Among these, motor vehicle accidents and asthma had the highest proportion of outpatient visits (6076 outpatient visits [90.0%] and 5877 outpatient visits [87.3%], respectively), followed by other respiratory end points. Cardiovascular end points had lower proportions of outpatient visits, with cerebrovascular diseases having the lowest (897 outpatient visits among 1917 inpatient or outpatient visits [25.3%]).

**Table 2.** Counts of ED Visit Outcomes

Outcome	Arizona		California		Utah	
	All ED visits, No.^a^	Outpatient visits, No. (%)	All ED visits, No.^a^	Outpatient visits, No. (%)	All ED visits, No.^a^	Outpatient visits, No. (%)
Asthma	3243	2768 (85.4)	3478	3100 (89.1)	13	9 (69.2)
COPD	2065	1274 (61.7)	1700	979 (57.6)	3	2 (66.7)
CNP	3122	1868 (59.8)	3166	2050 (64.8)	15	7 (46.7)
CHF	1349	467 (34.6)	1407	452 (32.1)	6	2 (33.3)
CVD	1765	604 (34.2)	1147	292 (25.5)	5	1 (20)
IHD	2536	901 (35.5)	1716	435 (25.3)	11	4 (36.4)
MVA	3621	3203 (88.5)	3076	2824 (91.8)	56	49 (87.5)

Abbreviations: CHF, congestive heart failure; COPD, chronic obstructive pulmonary disease; CNP, culture-negative pneumonia; CVD, cerebrovascular disease; ED, emergency department; IHD, ischemic heart disease; and MVA, motor vehicle accident.

^a^ Counts of ED visits during the study period are given for 3 southwestern US states: Arizona (July 1, 2010, to 2018), California (2005-2018), and Utah (2005-2016). Counts of specifically outpatient visits are included, as well as the percentage of all visits that were outpatient. Analysis was restricted to zip codes with at least a 5% area overlap with a National Weather Service forecast zone in which at least 1 dust storm was reported. Outcomes were based on the primary diagnosis, except for motor vehicle accidents, for which both primary and secondary diagnoses were used. Only patients whose ED visits fell on the day of the dust storm in that zip code, on a control day, or on subsequent lag days are included.

ED visits were nearly balanced between Arizona (17 701 visits [52.8%]) and California (15 690 visits [46.8%]), with Utah making only a small contribution (109 visits [0.3%]) owing to the small number of dust storms reported (Table 3) and the low population in its dust-impacted zip codes. State-level demographics are reported in eTable 2 in Supplement 1.

**Table 3.** Summary Statistics of Dust Storm Exposure and Air Pollution

Measure^a^	State			Overall
	Arizona (July 1, 2010, to 2018)	California (2005-2018)	Utah (2005-2016)	
Zip codes, No.	128	70	8	206
No. of dust days per year, mean (SD)	16.33 (3.95)	5.07 (2.24)	0.17 (0.41)	14.28 (3.70)
Temperature, mean (IQR), °C	30.49 (28.17 to 33.02)	18.56 (12.94 to 24.36)	22.16 (21.64 to 23.24)	25.25 (22.80 to 30.35)
Dew point, mean (IQR), °C	9.98 (7.69 to 13.40)	1.10 (−3.28 to 4.93)	−0.21 (−1.36 to 1.05)	6.12 (2.55 to 10.11)
O_3_, mean (IQR), ppb	52.76 (48.88 to 57.09)	49.29 (42.48 to 55.92)	57.87 (54.95 to 59.98)	52.00 (47.9 to 57.01)
NO_2_, mean (IQR), ppb	10.10 (8.22 to 12.1)	13.82 (10.67 to 16.72)	12.74 (11.64 to 13.96)	12.74 (11.64 to 13.96)
PM_2.5_, mean (IQR), μg/m^3^	4.29 (3.31 to 5.23)	5.61 (3.68 to 7.02)	6.06 (5.33 to 6.80)	4.70 (3.46 to 5.68)

Abbreviations: NO_2_, nitrogen dioxide; O_3_, ozone; PM_2.5_, fine particulate matter.

^a^ Summary statistics of dust storm exposure and air pollution within dust storm–affected zip code tabulation areas are given by state. Analysis was restricted to zip codes with at least a 5% overlap with a dust storm event reported by a National Weather Service forecast zone. Air pollutant exposures were computed as 3-day moving means and include only concentration data from 2005 to 2016 due to availability. Mean and IQR were first calculated within each zip code, and then the median was calculated across zip codes. All days within strata are included.

Counts of ED visits after dust storms but not control days by state and exposure window are presented in eTable 3 in Supplement 1. For example, there were 172 ED visits for asthma in Arizona on the day of dust storms but 1168 ED visits over the 0 to 7–day lag period.

### Dust Storm Exposure Analysis

As presented in Table 3, Arizona had the largest number of dust-impacted zip codes (128 zip codes), followed by California (70 zip codes) and Utah (8 zip codes). Among dust-impacted zip codes, Arizona also had the highest mean (SD) number of dust storm days per year (16.3 [4.0] days) followed by California (5.1 [2.2] days) and Utah (0.2 [0.4] days).

### Health Outcomes Associated With Dust Storms

As shown in the Figure, dust storms were positively associated with ED visits for asthma over the 0 to 2–day exposure window (RR, 1.06; 95% CI, 1.01-1.11; *P* = .03), culture-negative pneumonia (RR, 1.06; 95% CI, 1.02-1.10; *P* = .002) and congestive heart failure (RR, 1.06; 95% CI, 1.01-1.10; *P* = .01) over the 0 to 7–day window, and motor vehicle accidents on the day of the dust storm (RR, 1.13; 95% CI, 1.04-1.23; *P* = .003). However, dust storms were negatively associated with ED visits for culture-negative pneumonia on the day of the storm (RR = 0.85; 95% CI, 0.78-0.93; *P* < .001) and ischemic heart disease over 3 exposure windows (eg, 0-2 days: RR, 0.89; 95% CI, 0.84-0.95; *P* < .001). Crude estimates were similar to the main model estimates excepting positive associations with cerebrovascular disease at lags 0 and 0 to 5 days (eFigure 2 in Supplement 1).

**Figure.**
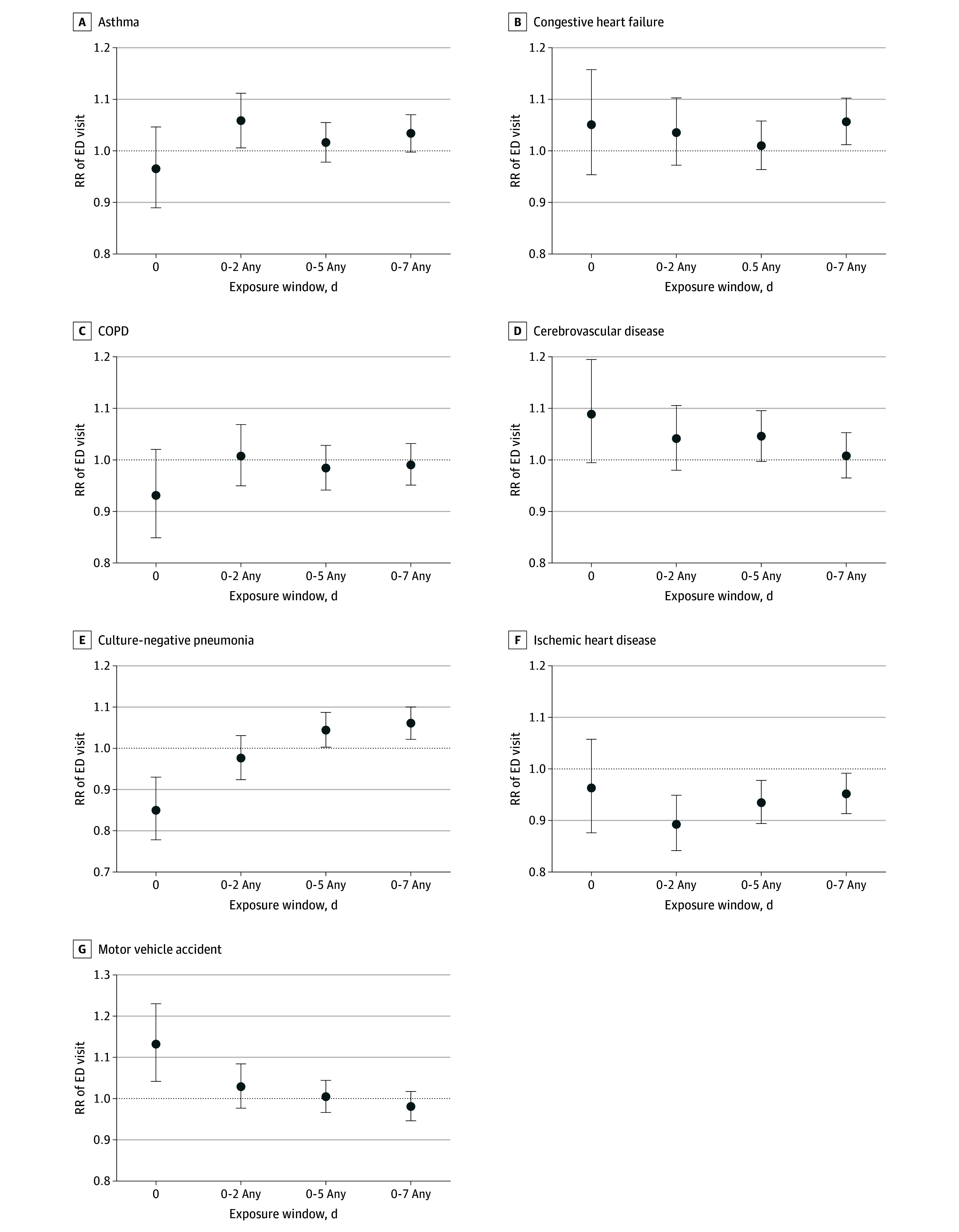
Association of Dust Storms With Emergency Department (ED) Visits by Health Outcome Dust storms could occur on any day of the given lag day range. COPD indicates chronic obstructive pulmonary disease; RR, relative risk; whiskers, 95% CIs.

### Attributable Fraction

The fraction of ED visits attributable to dust storms ranged as high as 1.79% (95% CI, 0.61% to 2.94%) for culture-negative pneumonia (0-7–day lag) and 1.70% (95% CI, 0.39% to 3.02%) for congestive heart failure (0-7–day lag) (eFigure 3 in Supplement 1). Because underlying associations were negative for certain outcomes and lag windows, attributable fractions can be negative (eg, −1.74%; 95% CI, −2.84% to −0.56% for ischemic heart disease at the 0-5–day lag).

### Sensitivity Analyses

Increasing the areal overlap required to assign a dust storm in a WFZ to a zip code diminished the number of dust-impacted zip codes and thus the number of ED visits to be analyzed. CIs of dust storm-ED visit associations were therefore wider (eFigure 4 in Supplement 1). Associations with culture-negative pneumonia remained positive for longer time windows when the threshold was set at 10% but not at 20%. Defining health outcomes using both primary and secondary *ICD-9* and *ICD-10* diagnosis codes, compared with use of primary diagnoses only, yielded some differences in observed associations (eFigure 5 in Supplement 1), including the attenuation of negative associations observed with ischemic heart disease and culture-negative pneumonia but stronger positive associations with cerebrovascular disease at lags 0 and 0 to 2 days. Changing the number of degrees of freedom per year to control for time (eFigure 6 in Supplement 1) and including air pollution covariates in the models (eFigure 7 in Supplement 1) produced results similar to the primary analyses. Associations with risk of ED visits for CHF and motor vehicle accidents were robust against adjustment for ambient O_3_(eg, CHF: RR, 1.08; 95% CI, 1.03-1.13; *P* = .003) and NO_2_ (eg, CHF: RR, 1.08; 95% CI, 1.03-1.13; *P* = .003) (eFigure 7 in Supplement 1). PM_2.5_ and O_3_ were positively associated with asthma. PM_2.5_ was also negatively associated with cerebrovascular disease, while NO_2_ was negatively associated with COPD. All 3 pollutants were negatively associated with ischemic heart disease (eFigure 8 in Supplement 1). Stratifying results by ED visits resulting in inpatient hospitalization vs outpatient visits yielded large differences for congestive heart failure and COPD at lags 0 and 0 to 2 days, with dust storms having a positive association with inpatient visits and a negative association with outpatient visits. Dust storms were negatively associated with inpatient visits for asthma at lag 0 days, but results were consistent with the null hypothesis for outpatient visits. Over lags 0 to 2 days, dust storms were positively associated with outpatient visits and inpatient visit outcomes were consistent with the null hypothesis. Dust storms were positively associated with ED visits for cerebrovascular disease at all lags and motor vehicle accidents at lag 0 only for outpatient visits (eFigure 9 in Supplement 1).

## Discussion

In this cross-sectional study of dust storms and ED visits in 3 Southwestern US states using a time-stratified case-crossover design, we observed robust associations of dust storms identified by NWS reports with ED visits for several health outcomes. In particular, dust storms were consistently and positively associated with ED visits for motor vehicle accidents on the day of the dust storm. Dust storms were also positively associated with asthma and congestive heart failure. There were no observed associations for other outcomes, and associations were negative for ischemic heart disease. These results were robust to changes in different confounder controls, and the positive association with motor vehicle accidents was robust to changes to the WFZ to zip code assignment.

Overall, the fraction of ED visits attributable to dust storms was modest. This may reflect the possibility that reports of dust storms included relatively small or low-intensity dust storms. Furthermore, reported storm durations in the database were overwhelmingly less than 3 hours and thus may have yielded small cumulative exposures to PM.

While we have used primary *ICD-9* and *ICD-10* diagnosis in the definition of health outcomes (excepting for motor vehicle accidents), the choice of whether to include secondary *ICD-9* or *ICD-10* diagnosis codes is not obvious. When we included both, our results tended to be attenuated toward the null except for cerebrovascular diseases.

Similarly, ED visit diagnoses vary in how frequently they result in inpatient hospital admission, which can obscure differences in mean severity between, for example, respiratory and cardiovascular diagnoses. A sensitivity analysis stratifying by inpatient vs outpatient visits found that dust storm associations for inpatient visits were more positive than for outpatient visit congestive heart failure and COPD, but more negative for asthma, cerebrovascular disease, ischemic heart disease, and motor vehicle accidents..

The findings of a positive association with asthma ED visits over the 0 to 2–day time window and with culture-negative pneumonia over the 0 to 7–day window are broadly consistent with results in the literature on respiratory outcomes associated with coarse PM.^79,80,81,82^ However, results for cardiovascular outcomes were null in most of our primary models but positive for cerebrovascular disease at lag 0 and lags 0 to 2 days when including secondary diagnosis *ICD-9* and *ICD-10* codes. This is in contrast to previous work, which found associations with cardiovascular mortality using primary *ICD-9* codes only^3^ and no associations with cardiovascular ICU admissions using primary and secondary *ICD-9* and *ICD-10* codes together.^57^ While ischemic heart disease tended to yield protective dust storm associations in our main models at lag 0 to 2 and 0 to 5 days, these associations were no longer apparent in the model with secondary *ICD-9* and *ICD-10* codes included. However, one explanation for a negative association may be that ischemic heart disease complications tend to be more severe on dust storm days, and therefore a higher fraction of patients would be admitted straight to the hospital without first going through the ED. Ischemic heart disease was already the most likely to be associated with subsequent hospital admission of any outcome we studied.

The positive association between dust storms and motor vehicle accidents observed here is in contrast to the protective association found previously using data from Phoenix, Arizona.^83^ However, the difference in the direction of association may be attributable to methodological differences. For instance, the prior work focused on a single metropolitan area rather than several states and compared ED visits during the 6 hours after the storm with those in the 6 hours prior to the storm rather than comparing daily ED counts across a month as we did. That work also used a different and likely stricter definition of a haboob than the dust storm definition used here, and it may be that individuals avoid driving in the worst due events but not the more moderate ones and thereby flip the sign of the association.

Dust and sand storms have been associated with motor vehicle accidents and associated health outcomes in a variety of regions, including California,^62^ Saudi Arabia,^60^ and southeastern Iran.^59^ In the US specifically, researchers found that dust storms were responsible for 232 motor vehicle accident deaths in the US over an 11-year period.^61^ However, null results were reported in Kuwait^84^ and Cape Province, South Africa.^85^ The differences may point to differences in dust storm characteristics, driving behavior, and the presence of warning systems for drivers.

### Limitations

To our knowledge, this is the largest and most comprehensive study to date of dust storms and ED visits in the US. However, it has several limitations. First, the accuracy of dust storm events listed in the NWS database has been justifiably questioned as being based on potentially inaccurate and subjective observations lacking in quality control,^86^ which can result in dust storms being improperly included or excluded. While efforts have been made to find a better dust characterization approach for health studies,^87^ there is no widely used alternative. Second, the assignment of these events (localized to WFZs, which tend to be relatively large) to zip codes that can be much smaller and do not often align with WFZ boundaries requires choosing how best to perform the assignment. While we followed a previously published approach,^57^ our sensitivity analysis results demonstrated how different choices regarding how to assign WFZs to zip codes led to qualitatively different RR estimates. Third, our work did not differentiate between larger WFZs, which may overlap more zip codes but yield greater exposure misclassification, and smaller WFZs, which may not. Fourth, the unit of analysis used here was the zip code–day, and thus our results were impacted by the ecologic fallacy whereby a uniform exposure is assigned to multiple individuals although true individual exposures vary. Fifth, we performed a large number of statistical tests, which heighten the chance of a false-positive result. Using a more stringent *P* value threshold of .01, positive associations were still evidenced for culture-negative pneumonia (lag 0-7) and motor vehicle accidents (lag 0) and negative associations for culture-negative pneumonia (lag 0) and ischemic heart disease (lag 0-2). Sixth, our focus on ED visits as a less severe outcome than hospitalizations by necessity fails to encompass direct admissions to the hospital, a common occurrence for acute cardiovascular problems. While it is not always clear whether or in which direction these limitations may have biased our results, we speculate that the accuracy of the database, mapping between WFZs and zip codes, and ecologic fallacy may have produced nondifferential exposure misclassification and thus biased our results toward the null.

## Conclusions

This cross-sectional study’s finding of the increase in motor vehicle accidents associated with dust storm days underscores the immediate dangers posed by reduced visibility and hazardous driving conditions during such events. This finding supports the intuitive notion that dust storms are associated with health through biological pathways and through their influence on environmental safety.

Enhancing public awareness about the risks associated with dust storms, especially on days when high dust activity is forecasted, may help at-risk populations take preventive actions, such as avoiding driving, staying indoors, using air purifiers, or wearing protective masks. Health care professionals could also be alerted to the increased likelihood of dust storm–related health issues during these times, which could improve response times and preparedness in emergency and primary care settings.

## Notes

Supplement 1. **eFigure 1.** Map of dust storm-impacted zip codes using the 5% overlap dust storm event definition **eTable 1.** Outcome-specific *ICD* codes and counts of ED visits by state **eTable 2.** Demographic table of ED visits by state in dust-impacted zip codes **eTable 3.** Counts of ED visits cause for each state and exposure window **eFigure 2.** Comparison of results between main model and crude model **eFigure 3.** Attributable fraction of ED visits due to dust storms in zip code-days under study **eFigure 4.** Short-term associations between ED visits and dust storm events, varied by different dust storm event definition (5%, 10%, 20%) **eFigure 5.** Short-term associations between ED visits and dust storm events, varied by primary versus primary and secondary outcome **eFigure 6.** Short-term associations between ED visits and dust storm events, varied by different temporal controls **eFigure 7.** Short-term associations between ED visits and dust storm events during 2005-2016, controlled for 3-days (lag 0-2) moving mean of ozone, NO2, PM2.5, separately and jointly **eFigure 8.** Short-term associations between ED visits and dust storm co-pollutants during 2005-2016 expressed in terms of a 1 IQR increase in the pollutant concentration **eFigure 9.** Short-term associations between ED visits and dust storm events stratified by ED visits resulting in inpatient hospitalizations vs. outpatient visits

Supplement 2.
Data Sharing Statement

## References

[1] Wu C, Lin Z, Shao Y, Liu X, Li Y. Drivers of recent decline in dust activity over East Asia. Nat Commun. 2022;13(1):7105. doi:10.1038/s41467-022-34823-3 36402787 PMC9675820

[2] Aryal Y, Evans S. Dust emission response to precipitation and temperature anomalies under different climatic conditions. Sci Total Environ. 2023;874:162335. doi:10.1016/j.scitotenv.2023.162335 36858225

[3] Crooks JL, Cascio WE, Percy MS, Reyes J, Neas LM, Hilborn ED. The association between dust storms and daily non-accidental mortality in the United States, 1993-2005. Environ Health Perspect. 2016;124(11):1735-1743. doi:10.1289/EHP216 27128449 PMC5089887

[4] East AE, Sankey JB. Geomorphic and sedimentary effects of modern climate change: current and anticipated future conditions in the Western United States. Rev Geophys. 2020;58(4):e2019RG000692. doi:10.1029/2019RG000692

[5] Shi L, Zhang J, Yao F, Zhang D, Guo H. Drivers to dust emissions over dust belt from 1980 to 2018 and their variation in two global warming phases. Sci Total Environ. 2021;767:144860. doi:10.1016/j.scitotenv.2020.144860 33434842

[6] Zong Q, Mao R, Gong DY, . Changes in dust activity in spring over East Asia under a global warming scenario. Asia Pac J Atmos Sci. 2021;57:839-850. doi:10.1007/s13143-021-00224-7

[7] Li J, Hao X, Liao H, . Predominant type of dust storms that influences air quality over northern China and future projections. Earth’s Future. 2022;10(6):e2022EF002649. doi:10.1029/2022EF002649

[8] Li Y, Mickley LJ, Kaplan JO. Response of dust emissions in southwestern North America to 21st century trends in climate, CO_2_ fertilization, and land use: implications for air quality. Atmos Chem Phys. 2021;21(1):57-68. doi:10.5194/acp-21-57-2021

[9] Zittis G, Almazroui M, Alpert P, Climate change and weather extremes in the eastern Mediterranean and Middle East. Rev Geophys. 2022;60(3):e2021RG000762. doi:10.1029/2021RG000762

[10] US Environmental Protection Agency. Supplement to the 2019 Integrated Science Assessment for Particulate Matter (Final Report, 2022). US Environmental Protection Agency; 2022. Accessed December 12, 2024. https://cfpub.epa.gov/ncea/isa/recordisplay.cfm?deid=354490 36630543

[11] US Environmental Protection Agency. Integrated Science Assessment (ISA) for Particulate Matter (Final Report, Dec 2019). US Environmental Protection Agency; 2019. Accessed December 12, 2024. https://assessments.epa.gov/isa/document/&deid=347534

[12] Yoo Y, Choung JT, Yu J, Kim DK, Koh YY. Acute effects of Asian dust events on respiratory symptoms and peak expiratory flow in children with mild asthma. J Korean Med Sci. 2008;23(1):66-71. doi:10.3346/jkms.2008.23.1.66 18303201 PMC2526497

[13] Yang CY, Chen YS, Chiu HF, Goggins WB. Effects of Asian dust storm events on daily stroke admissions in Taipei, Taiwan. Environ Res. 2005;99(1):79-84. doi:10.1016/j.envres.2004.12.009 16053931

[14] Watanabe M, Yamasaki A, Burioka N, . Correlation between Asian dust storms and worsening asthma in western Japan. Allergol Int. 2011;60(3):267-275. doi:10.2332/allergolint.10-OA-0239 21364309

[15] Watanabe M, Noma H, Kurai J, . Association of sand dust particles with pulmonary function and respiratory symptoms in adult patients with asthma in western Japan using light detection and ranging: a panel study. Int J Environ Res Public Health. 2015;12(10):13038-13052. doi:10.3390/ijerph121013038 26501307 PMC4627015

[16] Watanabe M, Noma H, Kurai J, . Decreased pulmonary function in school children in Western Japan after exposures to Asian desert dusts and its association with interleukin-8. Biomed Res Int. 2015;2015:583293. doi:10.1155/2015/583293 26060816 PMC4427824

[17] Watanabe M, Noma H, Kurai J, . Effect of Asian dust on pulmonary function in adult asthma patients in western Japan: a panel study. Allergol Int. 2016;65(2):147-152. doi:10.1016/j.alit.2015.10.002 26666479

[18] Ueda K, Shimizu A, Nitta H, Inoue K. Long-range transported Asian dust and emergency ambulance dispatches. Inhal Toxicol. 2012;24(12):858-867. doi:10.3109/08958378.2012.724729 23033999

[19] Nakamura T, Hashizume M, Ueda K, . Asian dust and pediatric emergency department visits due to bronchial asthma and respiratory diseases in Nagasaki, Japan. J Epidemiol. 2016;26(11):593-601. doi:10.2188/jea.JE20150309 27180931 PMC5083323

[20] Hong YC, Pan XC, Kim SY, . Asian dust storm and pulmonary function of school children in Seoul. Sci Total Environ. 2010;408(4):754-759. doi:10.1016/j.scitotenv.2009.11.015 19939437

[21] Lee H, Kim H, Honda Y, Lim Y-H, Yi S. Effect of Asian dust storms on daily mortality in seven metropolitan cities of Korea. Atmos Environ. 2013;79:510-517. doi:10.1016/j.atmosenv.2013.06.046

[22] Lee H, Honda Y, Lim Y-H, Guo YL, Hashizume M, Kim H. Effect of Asian dust storms on mortality in three Asian cities. Atmos Environ. 2014;89:309-317. doi:10.1016/j.atmosenv.2014.02.048

[23] Park JW, Lim YH, Kyung SY, . Effects of ambient particulate matter on peak expiratory flow rates and respiratory symptoms of asthmatics during Asian dust periods in Korea. Respirology. 2005;10(4):470-476. doi:10.1111/j.1440-1843.2005.00728.x 16135170

[24] Higashi T, Kambayashi Y, Ohkura N, . Effects of Asian dust on daily cough occurrence in patients with chronic cough: a panel study. Atmos Environ. 2014;92:506-513. doi:10.1016/j.atmosenv.2014.04.034

[25] Chen YS, Sheen PC, Chen ER, Liu YK, Wu TN, Yang CY. Effects of Asian dust storm events on daily mortality in Taipei, Taiwan. Environ Res. 2004;95(2):151-155. doi:10.1016/j.envres.2003.08.008 15147920

[26] Kwon HJ, Cho SH, Chun Y, Lagarde F, Pershagen G. Effects of the Asian dust events on daily mortality in Seoul, Korea. Environ Res. 2002;90(1):1-5. doi:10.1006/enrs.2002.4377 12359184

[27] Kurai J, Watanabe M, Noma H, . Estimation of the effects of heavy Asian dust on respiratory function by definition type. Genes Environ. 2017;39(1):25. doi:10.1186/s41021-017-0085-9 29118866 PMC5664575

[28] Higashi T, Kambayashi Y, Ohkura N, . Exacerbation of daily cough and allergic symptoms in adult patients with chronic cough by Asian dust: a hospital-based study in Kanazawa. Atmos Environ. 2014;97:537-543. doi:10.1016/j.atmosenv.2014.01.041

[29] Hashizume M, Kim Y, Ng CFS, . Health effects of Asian dust: a systematic review and meta-analysis. Environ Health Perspect. 2020;128(6):66001. doi:10.1289/EHP5312 32589456 PMC7319773

[30] Nakao M, Ishihara Y, Kim CH, Hyun IG. The impact of air pollution, including Asian sand dust, on respiratory symptoms and health-related quality of life in outpatients with chronic respiratory disease in Korea: a panel study. J Prev Med Public Health. 2018;51(3):130-139. doi:10.3961/jpmph.18.021 29886708 PMC5996190

[31] Lai LW, Cheng WL. The impact of air quality on respiratory admissions during Asian dust storm periods. Int J Environ Health Res. 2008;18(6):429-450. doi:10.1080/09603120802272227 19031147

[32] Chen CS, Chan YS, Liu TC. Tracheitis hospital admissions are associated with Asia dust storm. Int J Environ Health Res. 2022;32(6):1337-1343. doi:10.1080/09603123.2021.1879740 33508951

[33] Perez L, Tobias A, Querol X, . Coarse particles from Saharan dust and daily mortality. Epidemiology. 2008;19(6):800-807. doi:10.1097/EDE.0b013e31818131cf 18938653

[34] Stafoggia M, Zauli-Sajani S, Pey J, ; MED-PARTICLES Study Group. Desert dust outbreaks in Southern Europe: contribution to daily PM_10_ concentrations and short-term associations with mortality and hospital admissions. Environ Health Perspect. 2016;124(4):413-419. doi:10.1289/ehp.1409164 26219103 PMC4829979

[35] Karanasiou A, Moreno N, Moreno T, Viana M, de Leeuw F, Querol X. Health effects from Sahara dust episodes in Europe: literature review and research gaps. Environ Int. 2012;47:107-114. doi:10.1016/j.envint.2012.06.012 22796892

[36] Tobías A, Pérez L, Díaz J, . Short-term effects of particulate matter on total mortality during Saharan dust outbreaks: a case-crossover analysis in Madrid (Spain). Sci Total Environ. 2011;412-413:386-389. doi:10.1016/j.scitotenv.2011.10.027 22055453

[37] Mallone S, Stafoggia M, Faustini A, Gobbi GP, Marconi A, Forastiere F. Saharan dust and associations between particulate matter and daily mortality in Rome, Italy. Environ Health Perspect. 2011;119(10):1409-1414. doi:10.1289/ehp.1003026 21970945 PMC3230430

[38] Zauli Sajani S, Miglio R, Bonasoni P, . Saharan dust and daily mortality in Emilia-Romagna (Italy). Occup Environ Med. 2011;68(6):446-451. doi:10.1136/oem.2010.058156 21172793

[39] Faustini A, Alessandrini ER, Pey J, ; MED-PARTICLES study group. Short-term effects of particulate matter on mortality during forest fires in Southern Europe: results of the MED-PARTICLES Project. Occup Environ Med. 2015;72(5):323-329. doi:10.1136/oemed-2014-102459 25691696

[40] Trianti SM, Samoli E, Rodopoulou S, Katsouyanni K, Papiris SA, Karakatsani A. Desert dust outbreaks and respiratory morbidity in Athens, Greece. Environ Health. 2017;16(1):72. doi:10.1186/s12940-017-0281-x 28666479 PMC5493869

[41] Reyes M, Díaz J, Tobias A, Montero JC, Linares C. Impact of Saharan dust particles on hospital admissions in Madrid (Spain). Int J Environ Health Res. 2014;24(1):63-72. doi:10.1080/09603123.2013.782604 23544440

[42] Díaz J, Tobías A, Linares C. Saharan dust and association between particulate matter and case-specific mortality: a case-crossover analysis in Madrid (Spain). Environ Health. 2012;11(1):11. doi:10.1186/1476-069X-11-11 22401495 PMC3328290

[43] Díaz J, Linares C, Carmona R, . Saharan dust intrusions in Spain: health impacts and associated synoptic conditions. Environ Res. 2017;156:455-467. doi:10.1016/j.envres.2017.03.047 28412538

[44] Perez L, Tobías A, Querol X, . Saharan dust, particulate matter and cause-specific mortality: a case-crossover study in Barcelona (Spain). Environ Int. 2012;48:150-155. doi:10.1016/j.envint.2012.07.001 22935765

[45] Neisi A, Vosoughi M, Idani E, . Comparison of normal and dusty day impacts on fractional exhaled nitric oxide and lung function in healthy children in Ahvaz, Iran. Environ Sci Pollut Res Int. 2017;24(13):12360-12371. doi:10.1007/s11356-017-8853-4 28357800

[46] Khaniabadi YO, Daryanoosh SM, Amrane A, . Impact of Middle Eastern dust storms on human health. Atmos Pollut Res. 2017;8(4):606-613. doi:10.1016/j.apr.2016.11.005

[47] Al-Taiar A, Thalib L. Short-term effect of dust storms on the risk of mortality due to respiratory, cardiovascular and all-causes in Kuwait. Int J Biometeorol. 2014;58(1):69-77. doi:10.1007/s00484-012-0626-7 23329278

[48] Vodonos A, Friger M, Katra I, . The impact of desert dust exposures on hospitalizations due to exacerbation of chronic obstructive pulmonary disease. Air Qual Atmos Health. 2014;7:433-439. doi:10.1007/s11869-014-0253-z

[49] Vodonos A, Friger M, Katra I, . Individual effect modifiers of dust exposure effect on cardiovascular morbidity. PLoS One. 2015;10(9):e0137714. doi:10.1371/journal.pone.0137714 26381397 PMC4575174

[50] Rutherford S, Clark E, McTainsh G, Simpson R, Mitchell C. Characteristics of rural dust events shown to impact on asthma severity in Brisbane, Australia. Int J Biometeorol. 1999;42(4):217-225. doi:10.1007/s004840050108 10232058

[51] Aragnou E, Watt S, Nguyen Duc H, . Dust transport from inland Australia and its impact on air quality and health on the eastern coast of Australia during the February 2019 dust storm. Atmosphere (Basel). 2021;12(2):141. doi:10.3390/atmos12020141

[52] Barnett AG, Fraser JF, Munck L. The effects of the 2009 dust storm on emergency admissions to a hospital in Brisbane, Australia. Int J Biometeorol. 2012;56(4):719-726. doi:10.1007/s00484-011-0473-y 21786212

[53] Johnston F, Hanigan I, Henderson S, Morgan G, Bowman D. Extreme air pollution events from bushfires and dust storms and their association with mortality in Sydney, Australia 1994-2007. Environ Res. 2011;111(6):811-816. doi:10.1016/j.envres.2011.05.007 21601845

[54] Neophytou AM, Yiallouros P, Coull BA, . Particulate matter concentrations during desert dust outbreaks and daily mortality in Nicosia, Cyprus. J Expo Sci Environ Epidemiol. 2013;23(3):275-280. doi:10.1038/jes.2013.10 23423218

[55] Karimi SM, Pouran H, Majbouri M, Moradi-Lakeh M, Hakimian H. Saharan sand and dust storms and neonatal mortality: evidence from Burkina Faso. Sci Total Environ. 2020;729:139053. doi:10.1016/j.scitotenv.2020.139053 32498181

[56] Kanatani KT, Ito I, Al-Delaimy WK, Adachi Y, Mathews WC, Ramsdell JW; Toyama Asian Desert Dust and Asthma Study Team. Desert dust exposure is associated with increased risk of asthma hospitalization in children. Am J Respir Crit Care Med. 2010;182(12):1475-1481. doi:10.1164/rccm.201002-0296OC 20656941 PMC3159090

[57] Rublee CS, Sorensen CJ, Lemery J, . Associations between dust storms and intensive care unit admissions in the United States, 2000-2015. Geohealth. 2020;4(8):GH000260. doi:10.1029/2020GH000260 32783014 PMC7411550

[58] Gyan K, Henry W, Lacaille S, . African dust clouds are associated with increased paediatric asthma accident and emergency admissions on the Caribbean island of Trinidad. Int J Biometeorol. 2005;49(6):371-376. doi:10.1007/s00484-005-0257-3 15692817

[59] Miri A, Middleton N. Long-term impacts of dust storms on transport systems in south-eastern Iran. Nat Hazards (Dordr). 2022;114:291-312. doi:10.1007/s11069-022-05390-z

[60] Islam MM, Alharthi M, Alam MM. The impacts of climate change on road traffic accidents in Saudi Arabia. Climate (Basel). 2019;7(9):103. doi:10.3390/cli7090103

[61] Tong D, Feng I, Gill TE, Schepanski K, Wang J. How Many People Were Killed by Windblown Dust Events in the United States? Bull Am Meteorol Soc. 2023;104(5):E1067-E1084. doi:10.1175/BAMS-D-22-0186.1

[62] Bhattachan A, Okin GS, Zhang J, Vimal S, Lettenmaier DP. Characterizing the role of wind and dust in traffic accidents in California. Geohealth. 2019;3(10):328-336. doi:10.1029/2019GH000212 32159022 PMC7007095

[63] Singh A, Kumar DP, Shivaprasad K, Mohit M, Wadhawan A, eds. Vehicle detection and accident prediction in sand/dust storms. 2021 International Conference on Computing Sciences (ICCS). 2021:107-111. doi:10.1109/ICCS54944.2021.00029

[64] Mohebbi A, Green GT, Akbariyeh S, Yu F, Russo BJ, Smaglik EJ. Development of dust storm modeling for use in freeway safety and operations management: an Arizona case study. Transp Res Rec. 2019;2673(5):175-187. doi:10.1177/0361198119839978

[65] Comrie AC. No consistent link between dust storms and valley fever (coccidioidomycosis). Geohealth. 2021;5(12):GH000504. doi:10.1029/2021GH000504 34877441 PMC8628988

[66] Tong DQ, Wang JXL, Gill TE, Lei H, Wang B. Intensified dust storm activity and Valley fever infection in the Southwestern United States. Geophys Res Lett. 2017;44(9):4304-4312. doi:10.1002/2017GL073524 30166741 PMC6108409

[67] Rowan C, R D’Souza R, Zheng X, . Dust storms and cardiorespiratory emergency department visits in three Southwestern United States: application of a monitoring-based exposure metric. Environ Res Health. 2024;2(3):031003. doi:10.1088/2752-5309/ad5751 39015250 PMC11247357

[68] Nicholas PK, Breakey S, McKinnon S, Eddy EZ, Fanuele J, Starodub R. A CLIMATE: a tool for assessment of climate-change-related health consequences in the emergency department. J Emerg Nurs. 2021;47(4):532-542.e1. doi:10.1016/j.jen.2020.10.002 33280889

[69] Hess JJ, Heilpern KL, Davis TE, Frumkin H. Climate change and emergency medicine: impacts and opportunities. Acad Emerg Med. 2009;16(8):782-794. doi:10.1111/j.1553-2712.2009.00469.x 19673715

[70] Hammad KS, Arbon P, Gebbie K, Hutton A. Nursing in the emergency department (ED) during a disaster: a review of the current literature. Australas Emerg Nurs J. 2012;15(4):235-244. doi:10.1016/j.aenj.2012.10.005 23217657

[71] Rubin R. The costs of US emergency department visits. JAMA. 2021;325(4):333. doi:10.1001/jama.2020.26936 33496760

[72] Gordon JA, Billings J, Asplin BR, Rhodes KV. Safety net research in emergency medicine: proceedings of the Academic Emergency Medicine Consensus Conference on “The Unraveling Safety Net”. Acad Emerg Med. 2001;8(11):1024-1029. doi:10.1111/j.1553-2712.2001.tb01110.x 11691663

[73] Henderson S, Stacey CL, Dohan D. Social stigma and the dilemmas of providing care to substance users in a safety-net emergency department. J Health Care Poor Underserved. 2008;19(4):1336-1349. doi:10.1353/hpu.0.0088 19029756

[74] Di Somma S, Paladino L, Vaughan L, Lalle I, Magrini L, Magnanti M. Overcrowding in emergency department: an international issue. Intern Emerg Med. 2015;10(2):171-175. doi:10.1007/s11739-014-1154-8 25446540

[75] Lall R, Abdelnabi J, Ngai S, . Advancing the use of emergency department syndromic surveillance data, New York City, 2012-2016. Public Health Rep. 2017;132(1_suppl)(suppl):23S-30S. doi:10.1177/0033354917711183 28692384 PMC5676519

[76] Schramm PJ, Vaidyanathan A, Radhakrishnan L, Gates A, Hartnett K, Breysse P. Heat-related emergency department visits during the Northwestern heat wave—United States, June 2021. MMWR Morb Mortal Wkly Rep. 2021;70(29):1020-1021. doi:10.15585/mmwr.mm7029e1 34292925 PMC8297695

[77] National Centers for Environmental Information. Storm events database. National Oceanic and Atmospheric Administration. Accessed December 26, 2024. https://www.ncdc.noaa.gov/stormevents/faq.jsp

[78] Di Q, Wei Y, Shtein A, . Daily and annual PM2.5 concentrations for the contiguous United States, 1-km grids, v1 (2000-2016). Socioeconomic Data and Applications Center. Accessed December 20, 2024. https://earthdata.nasa.gov/data/catalog/sedac-ciesin-sedac-aqdh-dapm25-us-1km-1.10-1.10

[79] Host S, Larrieu S, Pascal L, . Short-term associations between fine and coarse particles and hospital admissions for cardiorespiratory diseases in six French cities. Occup Environ Med. 2008;65(8):544-551. doi:10.1136/oem.2007.036194 18056749

[80] Adar SD, Filigrana PA, Clements N, Peel JL. Ambient coarse particulate matter and human health: a systematic review and meta-analysis. Curr Environ Health Rep. 2014;1(3):258-274. doi:10.1007/s40572-014-0022-z 25152864 PMC4129238

[81] Zanobetti A, Schwartz J. The effect of fine and coarse particulate air pollution on mortality: a national analysis. Environ Health Perspect. 2009;117(6):898-903. doi:10.1289/ehp.0800108 19590680 PMC2702403

[82] Samoli E, Stafoggia M, Rodopoulou S, ; MED-PARTICLES Study Group. Associations between fine and coarse particles and mortality in Mediterranean cities: results from the MED-PARTICLES project. Environ Health Perspect. 2013;121(8):932-938. doi:10.1289/ehp.1206124 23687008 PMC3734494

[83] Henry MB, Mozer M, Rogich JJ, . Haboob dust storms and motor vehicle collision-related trauma in Phoenix, Arizona. West J Emerg Med. 2023;24(4):798-804. doi:10.5811/WESTJEM.59381 37527375 PMC10393452

[84] Al-Hemoud A, Al-Sudairawi M, Neelamanai S, Naseeb A, Behbehani W. Socioeconomic effect of dust storms in Kuwait. Arabian Journal of Geosciences. 2017;10(18). doi:10.1007/s12517-016-2816-9

[85] Nkosi V, Mathee A, Blesic S, . Exploring meteorological conditions and human health impacts during two dust storm events in Northern Cape Province, South Africa: findings and lessons learnt. Atmosphere (Basel). 2022;13(3):424. doi:10.3390/atmos13030424

[86] Ardon-Dryer K, Gill TE, Tong D. When is a dust storm not a dust storm: examining the reliability of the storm events database for assessing the incidence of dust storms in the USA. ESS Open Archive. Preprint posted online June 23, 2022. doi:10.1002/essoar.10511691.1

[87] Hohsfield K, Rowan C, D’Souza R, Ebelt S, Chang H, Crooks J. Evaluating data product exposure metrics for use in epidemiologic studies of dust storms. Geohealth. 2023;7(8):GH000824. doi:10.1029/2023GH000824 37637996 PMC10459620

