# Supplementary material for: Dust Storms and Emergency Department Visits in 3 Southwestern States Using NWS Storm Reports
Source: JAMA Netw Open. 2025 Feb 12;8(2):e2457666. doi: 10.1001/jamanetworkopen.2024.57666 (PMC11822549; doi:10.1001/jamanetworkopen.2024.57666)
Supplement: Supplement 1. — eFigure 1. Map of dust storm-impacted zip codes using the 5% overlap dust storm event definition eTable 1. Outcome-specific ICD codes and counts of ED visits by state eTable 2. Demographic table of ED visits by state in dust-impacted zip codes eTable 3. Counts of ED visits cause for each state and exposure window eFigure 2. Comparison of results between main model and crude model eFigure 3. Attributable fraction of ED visits due to dust storms in zip code-days under study eFigure 4. Short-term associations between ED visits and dust storm events, varied by different dust storm event definition (5%, 10%, 20%) eFigure 5. Short-term associations between ED visits and dust storm events, varied by primary versus primary and secondary outcome eFigure 6. Short-term associations between ED visits and dust storm events, varied by different temporal controls eFigure 7. Short-term associations between ED visits and dust storm events during 2005-2016, controlled for 3-days (lag 0-2) moving mean of ozone, NO2, PM2.5, separately and jointly eFigure 8. Short-term associations between ED visits and dust storm co-pollutants during 2005-2016 expressed in terms of a 1 IQR increase in the pollutant concentration eFigure 9. Short-term associations between ED visits and dust storm events stratified by ED visits resulting in inpatient hospitalizations vs. outpatient visits [file jamanetwopen-e2457666-s001.pdf]

Supplemental Online Content

Zheng X, Chang HH, Ebelt ST, D'Souza R, Hohsfield K, Crooks JL. Dust storms and emergency department visits in 3 Southwestern states using NWS storm reports. *JAMA Netw Open*. 2025;8(2):e2457666. doi:10.1001/jamanetworkopen.2024.57666

eFigure 1. Map of dust storm-impacted zip codes using the 5% overlap dust storm event definition

eTable 1. Outcome-specific *ICD* codes and counts of ED visits by state

eTable 2. Demographic table of ED visits by state in dust-impacted zip codes

eTable 3. Counts of ED visits cause for each state and exposure window

eFigure 2. Comparison of results between main model and crude model

eFigure 3. Attributable fraction of ED visits due to dust storms in zip code-days under study

eFigure 4. Short-term associations between ED visits and dust storm events, varied by different dust storm event definition (5%, 10%, 20%)

eFigure 5. Short-term associations between ED visits and dust storm events, varied by primary versus primary and secondary outcome

eFigure 6. Short-term associations between ED visits and dust storm events, varied by different temporal controls

eFigure 7. Short-term associations between ED visits and dust storm events during 2005-2016, controlled for 3-days (lag 0-2) moving mean of ozone, NO2, PM2.5, separately and jointly

eFigure 8. Short-term associations between ED visits and dust storm co-pollutants during 2005-2016 expressed in terms of a 1 IQR increase in the pollutant concentration

eFigure 9. Short-term associations between ED visits and dust storm events stratified by ED visits resulting in inpatient hospitalizations vs. outpatient visits

This supplemental material has been provided by the authors to give readers additional information about their work.

© 2025 Zheng X et al. *JAMA Network Open*.

eFigure 1. Map of dust storm-impacted ZIP Codes using the 5% overlap dust storm event definition.

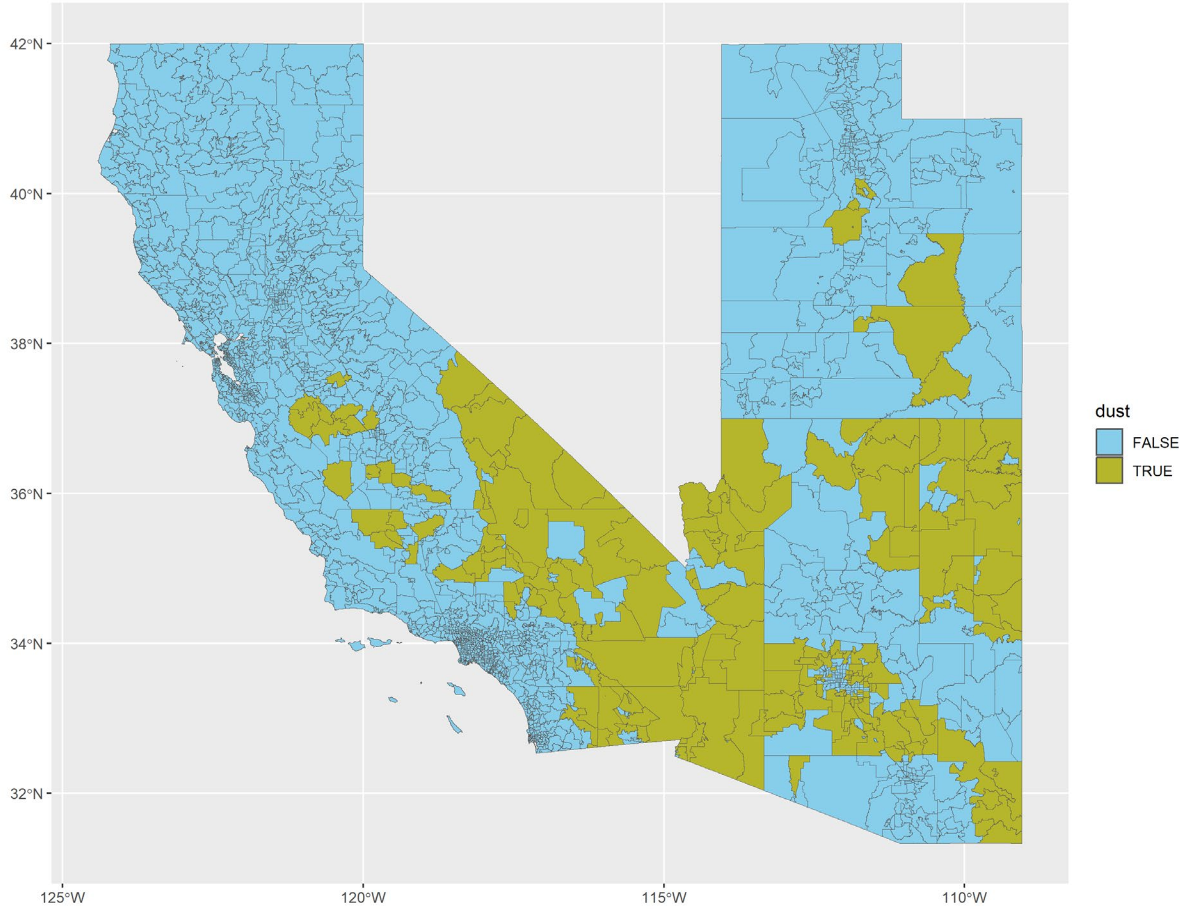

© 2025 Zheng X et al. *JAMA Network Open*.

eTable 1. Outcome-specific ICD codes and counts of ED visits by state.

Outcome	ICD-9 Codes	ICD-10 Codes	State<br>Arizona (2010-2018)	California (2005-2018)	Utah (2005-2016)	Total
Asthma	493	J45	17790	12977	32	30799
Chronic Obstructive Pulmonary Disease	491, 492, 496	J41 –J44	13816	8291	23	22130
Culture-negative Pneumonia	485, 486	J18	6170	5505	33	11708
Congestive Heart Failure	428	I42, I50-I51	10942	8100	39	19081
Cerebrovascular Disease	430-434, 436-438	I60-I69	4300	2531	11	6842
Ischemic Heart Disease	410-414	I20-I25	21478	10674	41	32193
Motor Vehicle Accident	E810-E819	V87-V89	3621	3076	56	6753

Outcome-specific ICD code ranges and counts of ED visits during the study period in three southwestern U.S. states: Arizona (7/1/2010-2018), California (2005-2018), and Utah (2005-2016). We restricted to only ZIP codes with at least a 5% area-overlap with a National Weather Service forecast zone. This table includes all ED visits during the period, not just those included in on or following dust storm and control days.

© 2025 Zheng X et al. *JAMA Network Open*.

eTable 2: Demographic table of ED visits by state in dust-impacted ZIP Codes

	AST				COPD				CNP				CHF				CVD				IHD				MVA			
**State**	**AZ**	**CA**	**UT**	**Total**	**AZ**	**CA**	**UT**	**Total**	**AZ**	**CA**	**UT**	**Total**	**AZ**	**CA**	**UT**	**Total**	**AZ**	**CA**	**UT**	**Total**	**AZ**	**CA**	**UT**	**Total**	**AZ**	**CA**	**UT**	**Total**
**Characteristic**	3,243	3,478	13	6,734	2,065	1,700	3	3,768	3,122	3,166	15	6,303	1,349	1,407	6	2,762	1,765	1,147	5	2,917	2,536	1,716	11	4,263	3,621	3,076	56	6,753
**Age**																												
**0-17**	1,318	1,596	6	2,920	1	0	0	1	546	1,115	0	1,661	2	1	0	3	10	9	1	20	0	1	0	1	608	485	18	1,111
**18-29**	524	525	2	1,051	9	10	0	19	183	197	2	382	7	8	0	15	16	14	0	30	9	8	0	17	1,075	1,042	18	2,135
**30-44**	582	552	3	1,137	50	58	0	108	389	323	2	714	85	71	0	156	106	57	0	163	131	74	0	205	898	702	5	1,605
**45-64**	534	565	1	1,100	761	752	2	1,515	704	618	4	1,326	377	425	1	803	534	322	0	856	1,064	687	5	1,756	754	602	8	1,364
**65 +**	253	240	1	494	1,244	880	1	2,125	1,178	913	7	2,098	873	902	5	1,780	1,097	745	4	1,846	1,332	946	6	2,284	271	245	7	523
**Unknown**	32	0	0	32	0	0	0	0	122	0	0	122	5	0	0	5	2	0	0	2	0	0	0	0	15	0	0	15
**Sex**																												
**Female**	1,725	1,718	4	3,447	1,095	975	1	2,071	1,503	1,504	6	3,013	535	597	4	1,136	826	540	3	1,369	902	663	2	1,567	1,893	1,569	39	3,501
**Male**	1,518	1,760	9	3,287	970	725	2	1,697	1,619	1,662	9	3,290	814	810	2	1,626	938	607	2	1,547	1,634	1,052	9	2,695	1,728	1,507	17	3,252
**Unknown**	0	0	0	0	0	0	0	0	0	0	0	0	0	0	0	0	1	0	0	1	0	1	0	1	0	0	0	0
**Race**																												
**Black**	604	455	0	1,059	94	126	0	220	217	152	0	369	130	90	0	220	111	58	0	169	118	79	0	197	419	176	0	595
**White**	1,420	2,265	11	3,696	1,764	1,399	3	3,166	1,961	2,323	15	4,299	904	1,125	3	2,032	1,242	925	5	2,172	1,913	1,394	8	3,315	1,702	2,110	45	3,857
**Other**	1,196	698	0	1,894	202	147	0	349	917	634	0	1,551	310	169	0	479	396	146	0	542	489	212	0	701	1,445	705	0	2,150
**Unknown**	23	60	2	85	5	28	0	33	27	57	0	84	5	23	3	31	16	18	0	34	16	31	3	50	55	85	11	151
**Ethnicity**																												
**Hispanic**	833	1,658	0	2,491	154	226	0	380	610	1,374	2	1,986	235	382	0	617	281	313	1	595	372	425	0	797	988	1,398	4	2,390
**Non-Hispanic**	2,386	1,757	11	4,154	1,899	1,440	3	3,342	2,487	1,735	13	4,235	1,108	1,007	3	2,118	1,472	818	4	2,294	2,144	1,267	8	3,419	2,573	1,584	42	4,199
**Unknown**	24	63	2	89	12	34	0	46	25	57	0	82	6	18	3	27	12	16	0	28	20	24	3	47	60	94	10	164

State-specific demographic table of ED visit patients in during the study period for three southwestern U.S. states: Arizona (7/1/2010-2018), California (2005-2018), and Utah (2005-2016). We restricted to only ZIP codes with at least a 5% area-overlap with a National Weather Service forecast zone in which at least one dust storm was reported. Outcomes were based on the primary diagnosis, except for motor vehicle accidents for which both primary and secondary diagnoses were used. AST = asthma, COPD = chronic obstructive pulmonary disease, CNP = culture-negative pneumonia, CVD = cerebrovascular disease, IHD = ischemic heart disease, and MVA = motor vehicle accidents.

© 2025 Zheng X et al. *JAMA Network Open*.

eTable 3: Counts of ED visits cause for each state and exposure window. ED visits following dust storms are included but ED visits following control days are not.

Counts of ED visits in dust-impacted ZIP Codes during exposed days, by exposure windows (days)	Arizona (7/1/2010-2018)				California (2005-2018)				Utah (2005-2016)			
Outcome	0	0-2	0-5	0-7	0	0-2	0-5	0-7	0	0-2	0-5	0-7
Asthma	172	497	901	1168	126	407	750	976	0	0	0	2
Chronic Obstructive Pulmonary Disease	107	293	554	708	68	190	348	457	0	0	0	0
Culture-negative Pneumonia	141	449	879	1122	116	332	650	859	1	4	5	6
Congestive Heart Failure	73	193	353	475	58	152	293	394	1	1	1	3
Cerebrovascular Disease	112	295	526	664	58	131	261	334	0	0	0	0
Ischemic Heart Disease	142	376	724	924	52	152	319	422	0	1	3	3
Motor Vehicle Accident	201	523	981	1233	114	306	614	811	1	8	16	20

© 2025 Zheng X et al. *JAMA Network Open*.

eFigure 2. Comparison of results between main model and crude model

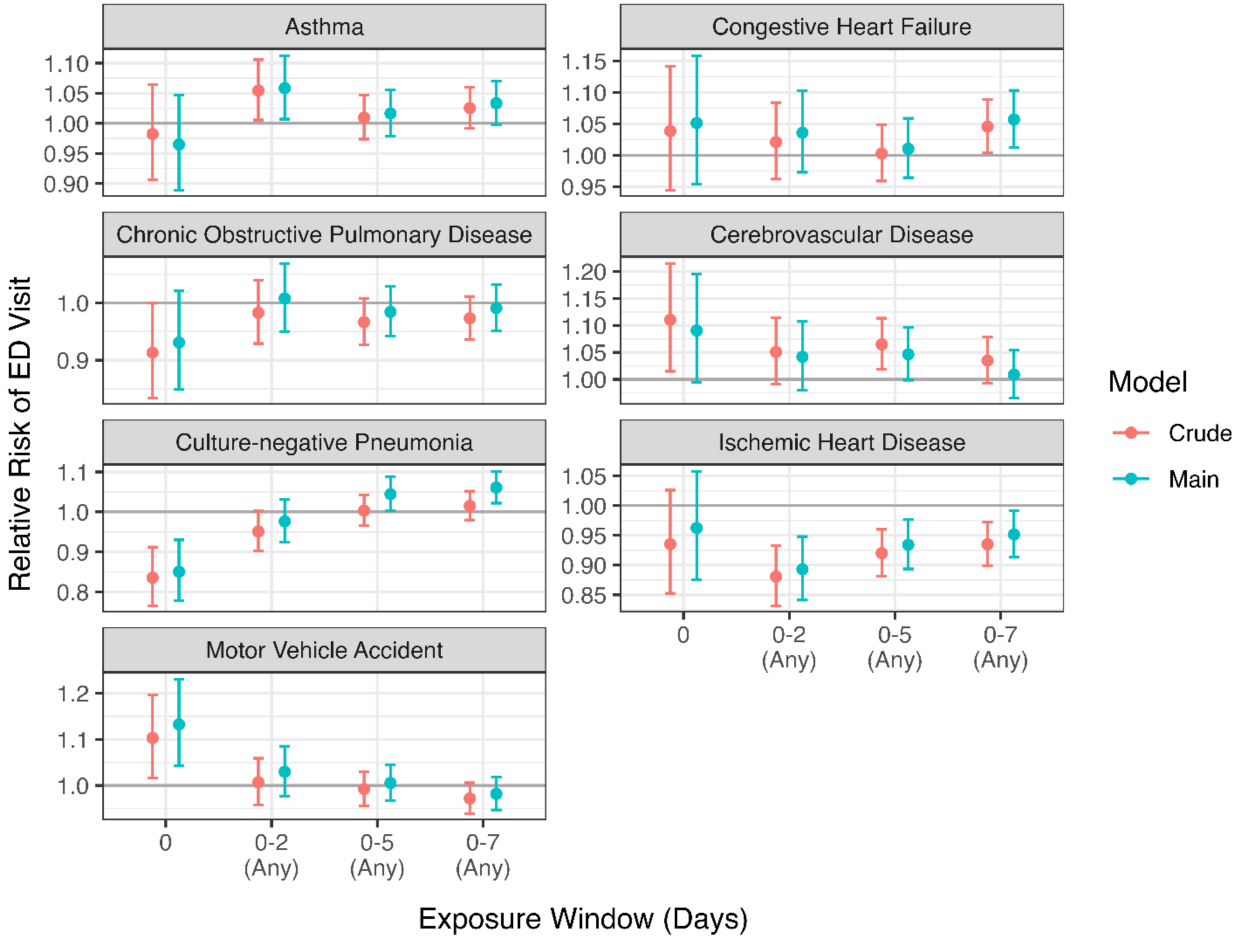

© 2025 Zheng X et al. *JAMA Network Open*.

eFigure 3. Attributable fraction of ED visits due to dust storms in ZIP Code-days under study.

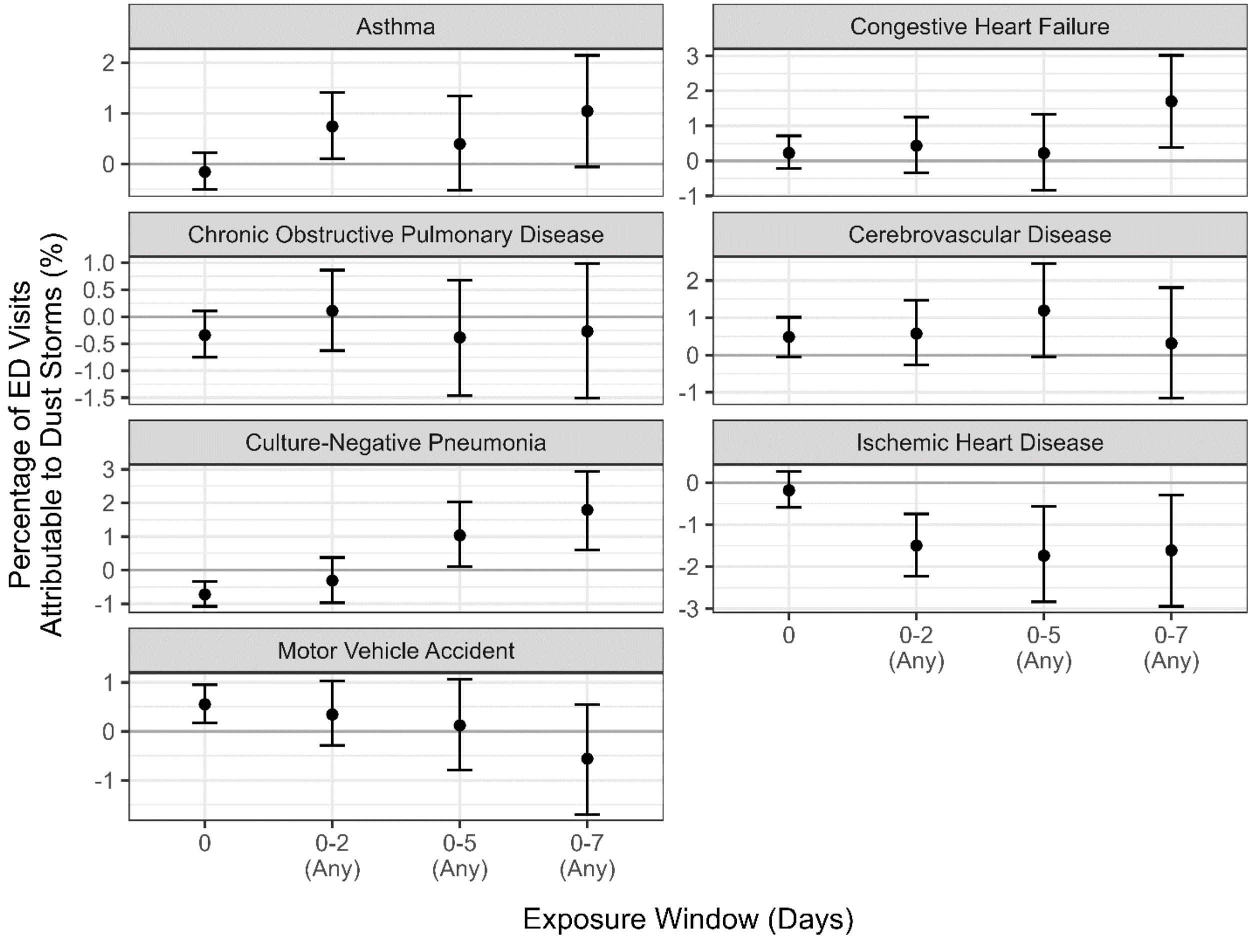

© 2025 Zheng X et al. *JAMA Network Open*.

eFigure 4. Short-term associations between ED visits and dust storm events, varied by different dust storm event definition (5%, 10%, 20%).

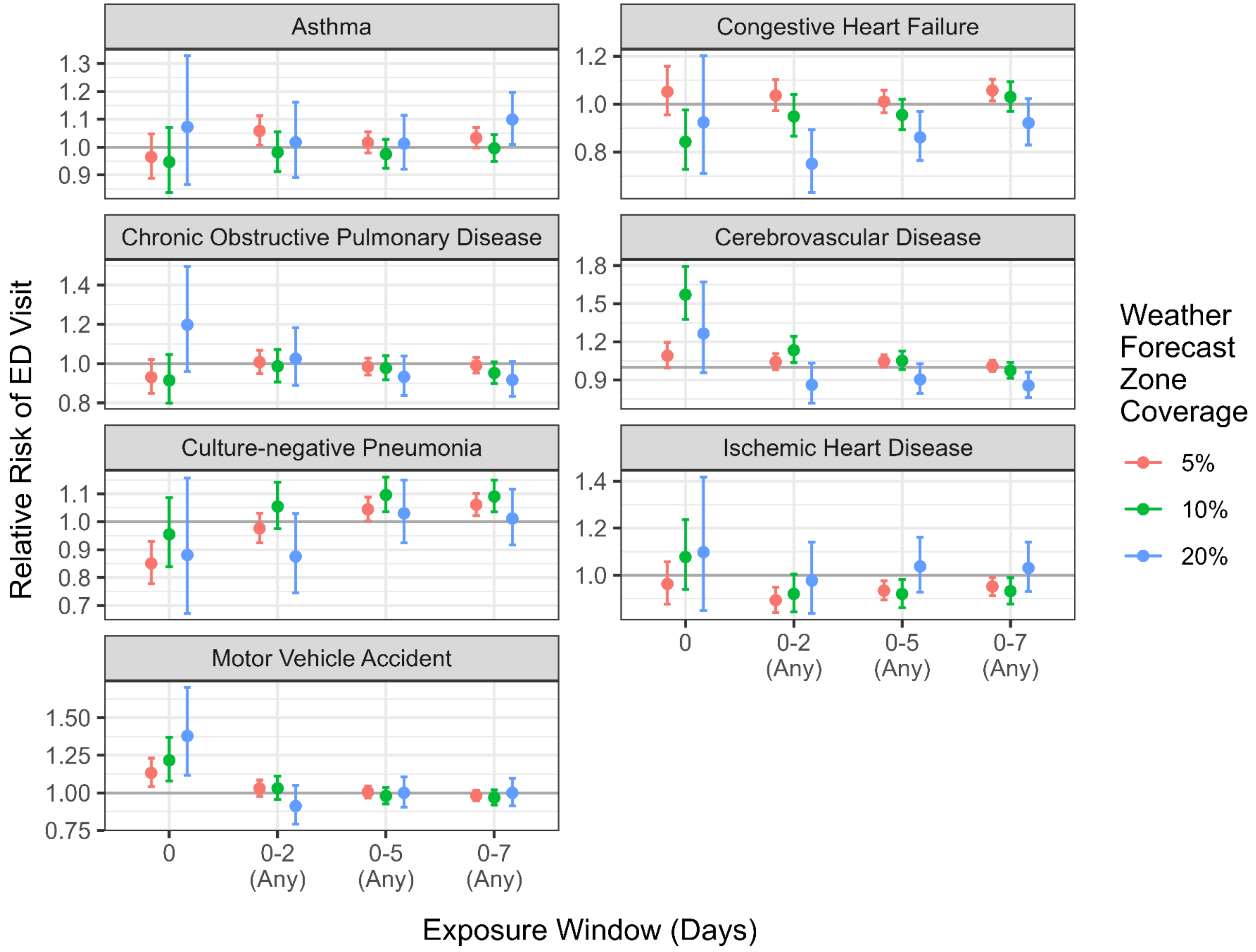

© 2025 Zheng X et al. *JAMA Network Open*.

eFigure 5. Short-term associations between ED visits and dust storm events, varied by primary versus primary and secondary outcome.

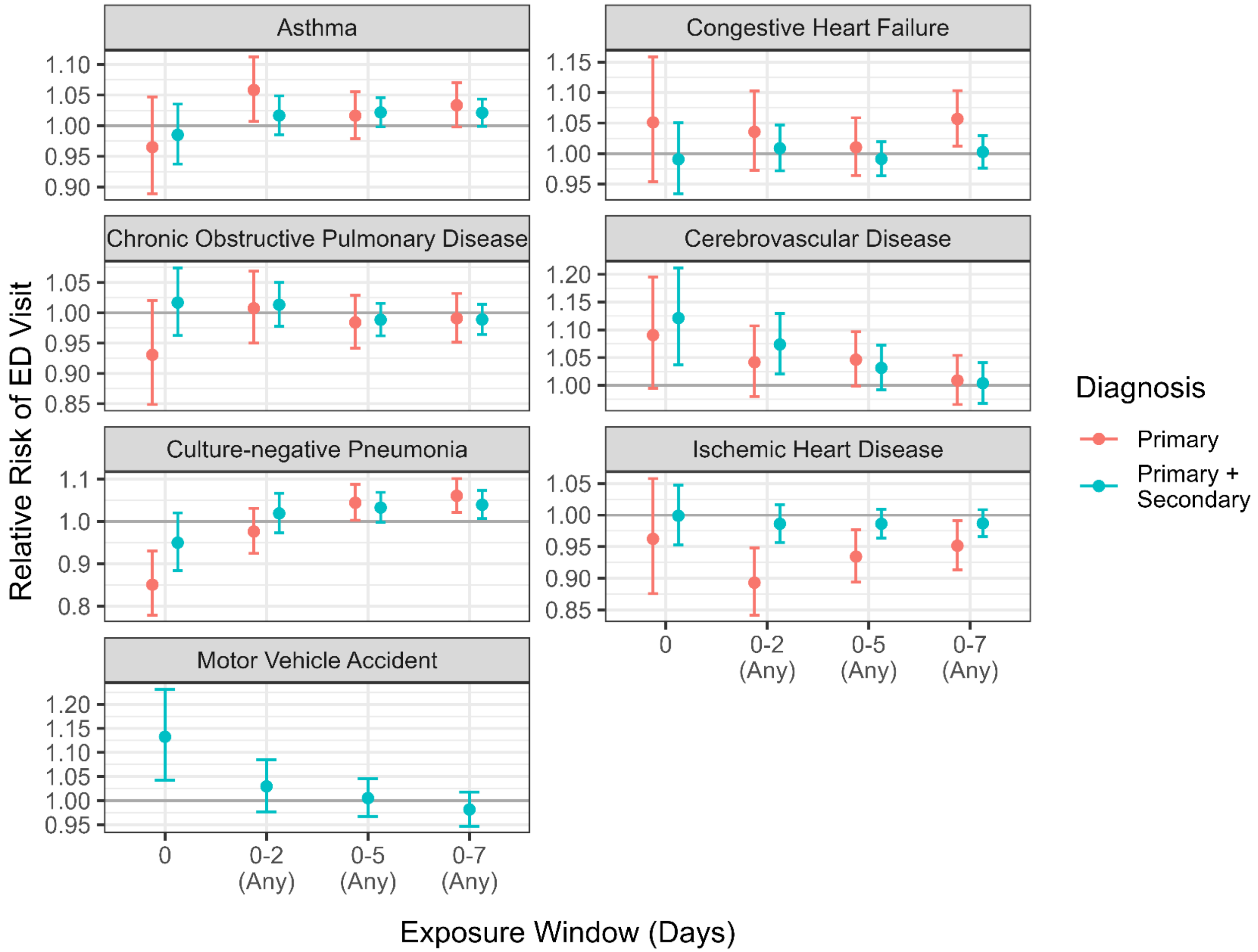

© 2025 Zheng X et al. *JAMA Network Open*.

eFigure 6. Short-term associations between ED visits and dust storm events, varied by different temporal controls.

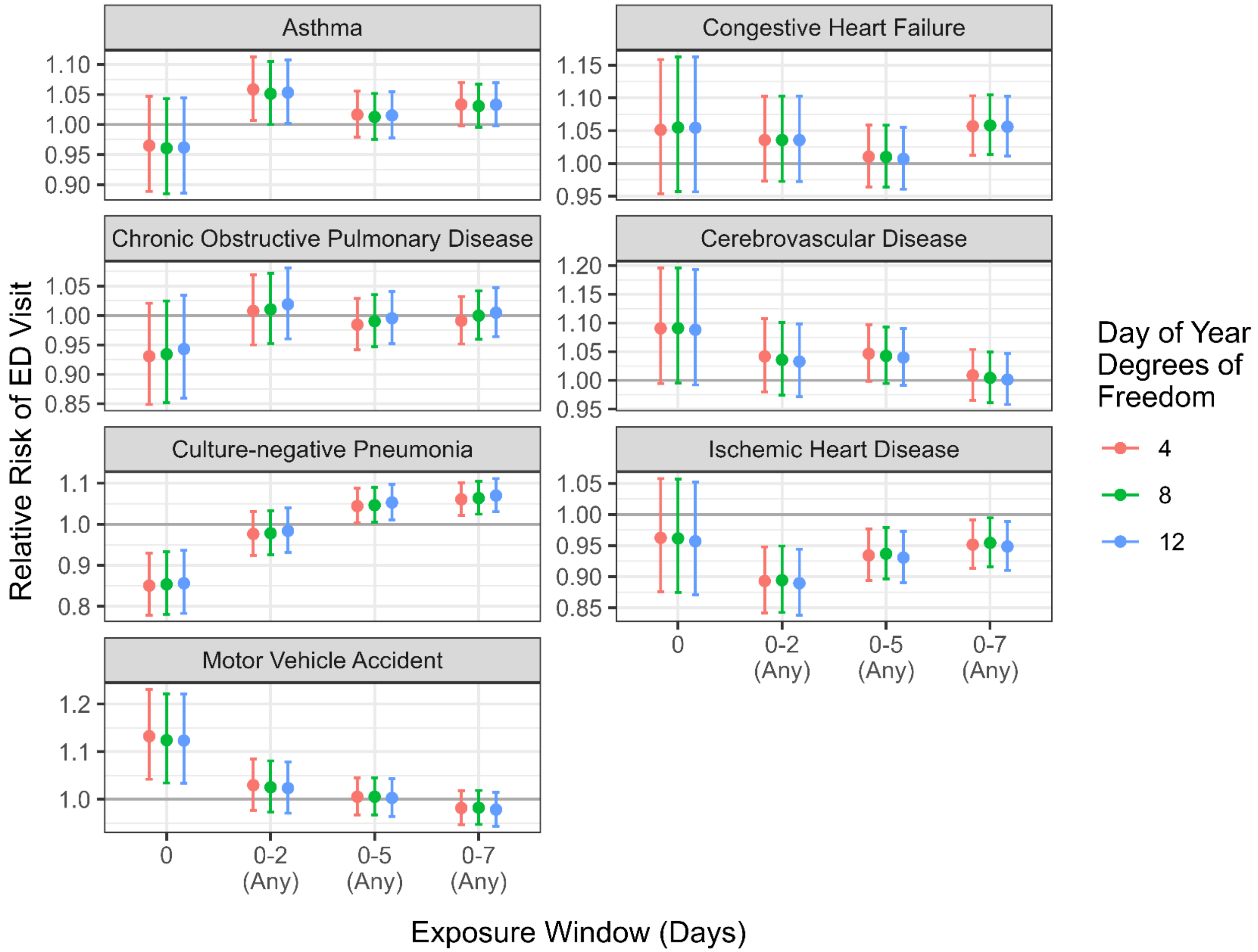

© 2025 Zheng X et al. *JAMA Network Open*.

eFigure 7. Short-term associations between ED visits and dust storm events during 2005-2016, controlled for 3-days (lag 0-2) moving average of Ozone, NO_2, PM2.5, separately and jointly. Only data through 2016 are included.

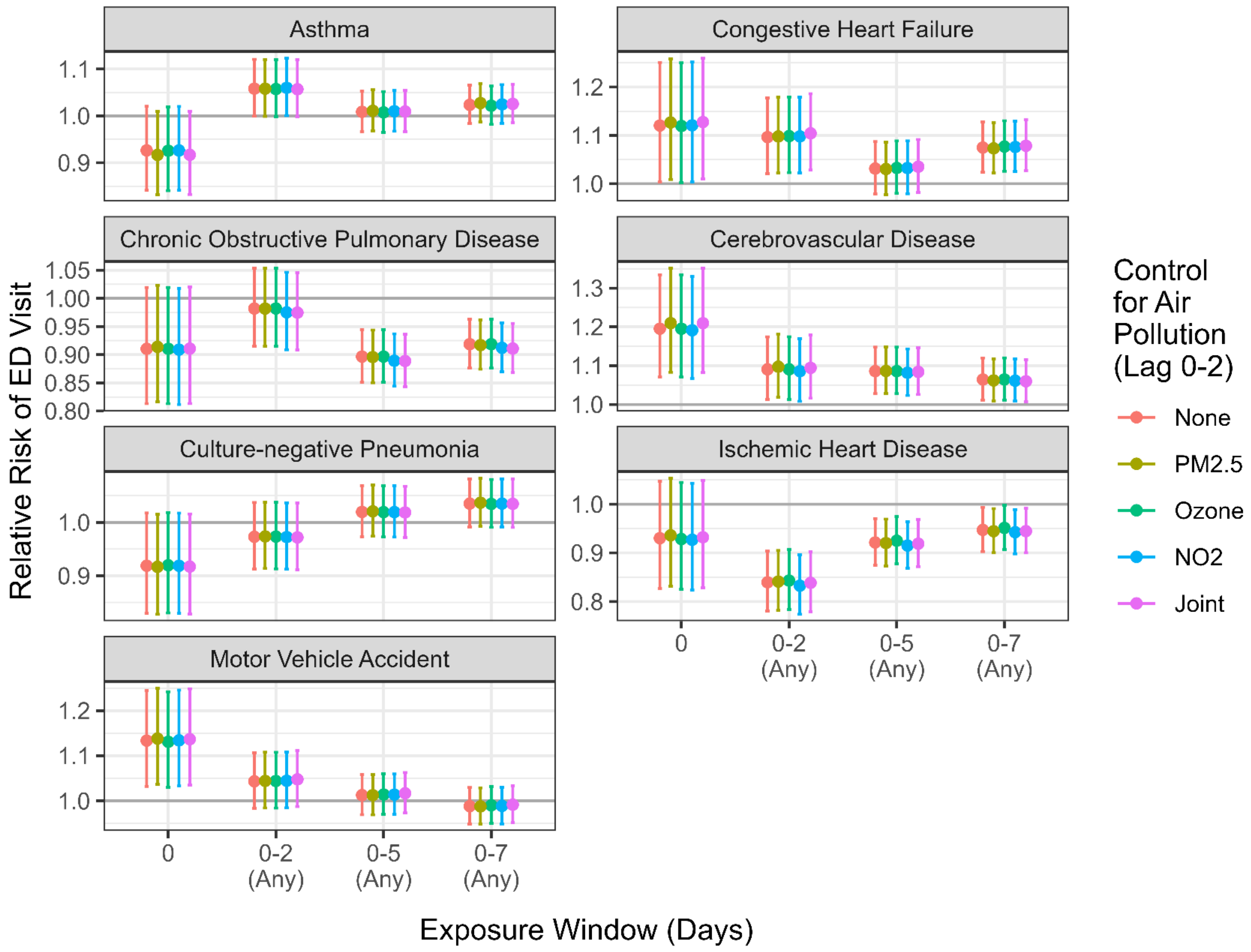

© 2025 Zheng X et al. *JAMA Network Open*.

eFigure 8. Short-term associations between ED visits and dust storm co-pollutants during 2005-2016 expressed in terms of a 1 inter-quartile range (IQR) increase in the pollutant concentration. The PM2.5, Ozone, and NO2 associations were estimated from models including only the given co-pollutant. The Joint association was estimated from a model including all three co-pollutants and was calculated by a linear combination of the co-pollutant-specific coefficients with the co-pollutant IQRs, with the resulting linear combination exponentiated to be presented on the relative risk scale.

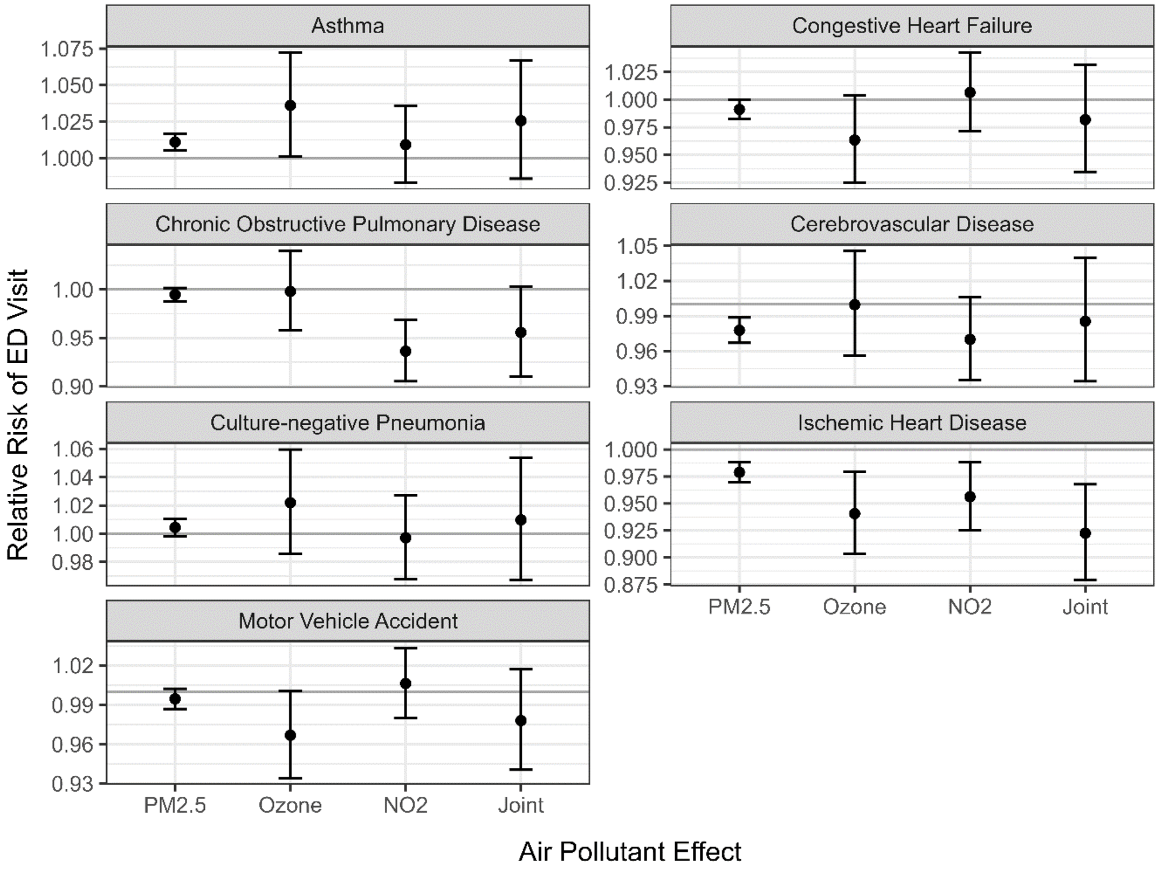

© 2025 Zheng X et al. *JAMA Network Open*.

eFigure 9. Short-term associations between ED visits and dust storm events stratified by ED visits resulting in inpatient hospitalizations vs. outpatient visits.

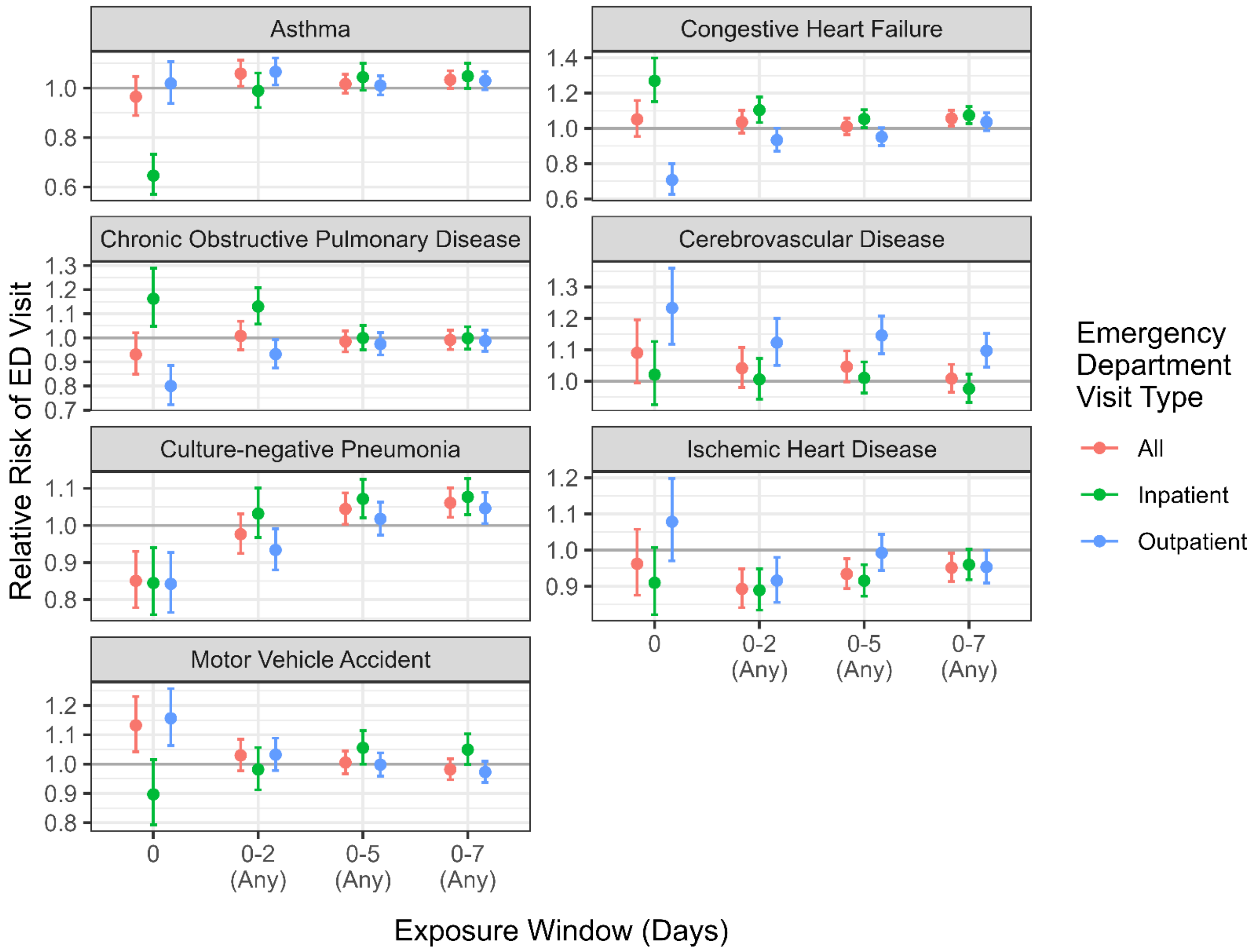

© 2025 Zheng X et al. *JAMA Network Open*.
