# Supplementary material for: Dust Storms and Emergency Department Visits in 3 Southwestern States Using NWS Storm Reports
Source: JAMA Netw Open. 2025 Feb 12;8(2):e2457666. doi: 10.1001/jamanetworkopen.2024.57666 (PMC11822549; doi:10.1001/jamanetworkopen.2024.57666)
Supplement: Supplement 2. — Data Sharing Statement [file jamanetwopen-e2457666-s002.pdf]

Data Sharing Statement

Zheng. Dust Storms and Emergency Department Visits in 3 Southwestern States Using NWS Storm Reports. *JAMA Netw Open*. Published online February 3, 2025. doi:10.1001/jamanetworkopen.2024.57666

Data
Data available: No

Additional Information
Explanation for why data not available: Exposure data are publicly available from the U.S. National Weather Service and NASA SEDAC. Emergency department visits were procured from state health departments under data use agreements and may not be shared. However, other researchers may procure these data by contacting the health departments.
